# Risk of conversion to mild cognitive impairment or dementia among subjects with amyloid and tau pathology: a systematic review and meta-analysis

**DOI:** 10.1186/s13195-024-01455-2

**Published:** 2024-04-12

**Authors:** Zsolt Huszár, Marie Anne Engh, Márk Pavlekovics, Tomoya Sato, Yalea Steenkamp, Bernard Hanseeuw, Tamás Terebessy, Zsolt Molnár, Péter Hegyi, Gábor Csukly

**Affiliations:** 1 Centre for Translational Medicine, Semmelweis University, Üllői út 26, Budapest, 1085 Hungary; 2 Department of Psychiatry and Psychotherapy, Semmelweis University, Balassa utca 6, Budapest, 1083 Hungary; 3 Department of Neurology, Jahn Ferenc Teaching Hospital, Köves utca 1, Budapest, 1204 Hungary; 4 Department of Neurology and Institute of Neuroscience, Cliniques Universitaires Saint-Luc, Université Catholique de Louvain, Brussels, 1200 Belgium; 5 Department of Radiology, Gordon Center for Medical Imaging, Massachusetts General Hospital, Harvard Medical School, Boston, MA 02155 USA; 6 Department of Anesthesiology and Intensive Therapy, Semmelweis University, Üllői út 78/A, Budapest, Hungary; 7 Department of Anesthesiology and Intensive Therapy, Poznan University of Medical Sciences, 49 Przybyszewskiego St, Poznan, Poland; 8 Institute for Translational Medicine, Medical School, University of Pécs, Pécs, 7624 Hungary; 9 Institute of Pancreatic Diseases, Semmelweis University, Tömő 25-29, Budapest, 1083 Hungary; 10 Translational Pancreatology Research Group, Interdisciplinary Centre of Excellence for Research Development and Innovation University of Szeged, Budapesti 9, Szeged, 6728 Hungary

**Keywords:** Beta-amyloid, Phosphorylated tau, Dementia, Mild cognitive impairment, Alzheimer’s disease

## Abstract

**Background:**

Measurement of beta-amyloid (Aβ) and phosphorylated tau (p-tau) levels offers the potential for early detection of neurocognitive impairment. Still, the probability of developing a clinical syndrome in the presence of these protein changes (A+ and T+) remains unclear. By performing a systematic review and meta-analysis, we investigated the risk of mild cognitive impairment (MCI) or dementia in the non-demented population with A+ and A- alone and in combination with T+ and T- as confirmed by PET or cerebrospinal fluid examination.

**Methods:**

A systematic search of prospective and retrospective studies investigating the association of Aβ and p-tau with cognitive decline was performed in three databases (MEDLINE via PubMed, EMBASE, and CENTRAL) on January 9, 2024. The risk of bias was assessed using the Cochrane QUIPS tool. Odds ratios (OR) and Hazard Ratios (HR) were pooled using a random-effects model. The effect of neurodegeneration was not studied due to its non-specific nature.

**Results:**

A total of 18,162 records were found, and at the end of the selection process, data from 36 cohorts were pooled (*n*= 7,793). Compared to the unexposed group, the odds ratio (OR) for conversion to dementia in A+ MCI patients was 5.18 [95% CI 3.93; 6.81]. In A+ CU subjects, the OR for conversion to MCI or dementia was 5.79 [95% CI 2.88; 11.64]. Cerebrospinal fluid Aβ42 or Aβ42/40 analysis and amyloid PET imaging showed consistent results. The OR for conversion in A+T+ MCI subjects (11.60 [95% CI 7.96; 16.91]) was significantly higher than in A+T- subjects (2.73 [95% CI 1.65; 4.52]). The OR for A-T+ MCI subjects was non-significant (1.47 [95% CI 0.55; 3.92]). CU subjects with A+T+ status had a significantly higher OR for conversion (13.46 [95% CI 3.69; 49.11]) than A+T- subjects (2.04 [95% CI 0.70; 5.97]). Meta-regression showed that the ORs for Aβ exposure decreased with age in MCI. (beta = -0.04 [95% CI -0.03 to -0.083]).

**Conclusions:**

Identifying Aβ-positive individuals, irrespective of the measurement technique employed (CSF or PET), enables the detection of the most at-risk population before disease onset, or at least at a mild stage. The inclusion of tau status in addition to Aβ, especially in A+T+ cases, further refines the risk assessment. Notably, the higher odds ratio associated with Aβ decreases with age.

**Trial registration:**

The study was registered in PROSPERO (ID: CRD42021288100).

**Supplementary Information:**

The online version contains supplementary material available at 10.1186/s13195-024-01455-2.

## Background

Affecting 55 million people worldwide, dementia is one of the leading causes of years spent with disability and one of the costliest long-term illnesses in society. The most common cause of dementia is Alzheimer's disease (AD), responsible for 60-80% of cases [1, 2].

Two specific protein aggregates play a crucial role in the pathophysiology of AD. One is the amyloid plaque formation in the extracellular space, predominantly by Aβ aggregation. These plaques, among other pathological effects, inhibit the signaling function of neurons [3]. The other protein change is the appearance of neurofibrillary tangles within the neurons, which are formed by the phosphorylation of tau proteins (p-tau) and inhibit the axonal transport inside the cell [4]. Whereas the specific pathology could only be confirmed by autopsy in the past, in vivo tests are available today. Parallelly to this development, the diagnostic definitions of AD have evolved significantly over time, moving from purely clinical assessments and post-mortem examinations to the integration of in vivo amyloid and later p-tau biomarkers, emphasizing the role of preclinical stages [5–8]. Accordingly, researchers are increasingly trying to link the diagnosis of the disease to biological parameters. However, in general, the clinical practice only considers the quality of the symptoms of dementia and the fact of neurodegeneration confirmed by radiology when establishing an AD diagnosis.

The International Working Group (IWG) [5] emphasizes that diagnosis should align with clinical symptoms. However, for researchers in the field, the U.S. National Institute on Aging – Alzheimer’s Association (NIA-AA) has issued a new framework recommendation [6]. This recommendation defines AD purely in terms of specific biological changes based on the Aβ (A) and p-tau (T) protein status, while neurodegeneration (N) is considered a non-specific marker that can be used for staging. In the recommendation, the category ‘Alzheimer’s disease continuum’ is proposed for all A+ cases, ‘Alzheimer’s pathological changes’ for A+T- cases, and ‘Alzheimer’s disease’ for A+T+ cases. A-(TN)+ cases are classified as ‘non-Alzheimer pathological changes’.

Aβ and p-tau proteins have long been known to be associated with AD development, and their accumulation can begin up to 15-20 years before the onset of cognitive symptoms [9]. Pathological amyloid changes are highly prevalent in dementia: 88% of those clinically diagnosed with AD and between 12 and 51% of those with non-AD are A+, according to a meta-analysis [10]. At the same time, the specificity of the abnormal beta-amyloid level for AD and its central role in its pathomechanism have been questioned [11]. Their use as a preventive screening target is a subject of ongoing discourse [12]. Yet it is still unclear to what extent their presence accelerates cognitive decline. What are the predictive prospects for an individual with abnormal protein levels who is otherwise cognitively healthy or with only mild cognitive impairment (MCI), meaning cases where there is a detectable decline in cognitive ability with maintained ability to perform most activities of daily living independently? [13] Research on non-demented populations shows substantial variation; for example, studies have shown OR values for conversion to dementia ranging from 2.25 [95% CI 0.71; 7.09] [14] to 137.5 [95% CI 17.8; 1059.6] [15]. Comparing conversion data systematically is necessary to provide a clearer picture.

In the CU population over 50 years, the prevalence of being A+ ranges from 10 to 44%, while in MCI it ranges from 27 to 71%, depending on age. Taking this into consideration [16], we aim to investigate the effect of Aβ alone and in combination with p-tau on the conversion to MCI and dementia, through a systematic review and meta-analysis of the available literature. Knowing the prognostic effect can highlight the clinical potential of this current research framework, given that, at present, the therapy of MCI or dementia can only slow down the decline. Prevention starting at an early stage or even before symptoms appear, provides the best chance against the disease.

## Methods

### Study registration

Our study was registered in the PROSPERO database (ID: CRD42021288100), with a pre-defined research plan and detailed objectives, is reported strictly in accordance with the recommendation of the PRISMA 2020 guideline and was performed following the guidance of the Cochrane Handbook [17].

We aimed to determine the change in odds of progression to MCI or dementia among non-demented subjects based on abnormal Aβ levels alone, or in combination with abnormal p-tau levels.

### Search and selection

We included longitudinal prospective and retrospective studies that used the NIA-AA 2018 recommended measurement of Aβ and p-tau (for Aβ: amyloid PET, CSF Aβ42, or Aβ42/40 ratio; for p-tau: tau PET, or CSF p-tau) and investigated the role of Aβ and +/- p-tau in CU and MCI subjects in progression to MCI or dementia. Case reports and case series were excluded. Overlapping populations were taken into account during the data extraction. Our search key was run in the Medline, Embase, and Central databases on 31 October 2021, and the search was updated on 9 January 2024 (see Supplementary Material, Appendix 1). After removing duplicates, we screened publications by title and abstract, and in the second round by full text. Two independent reviewers conducted the selection (ZH, MP), and a third reviewer (GC) resolved disagreements. The degree of the agreement was quantified using Cohen’s kappa statistics at each selection stage.

As part of the selection process, articles that only examined the ADNI database [18] were excluded, as patient-level data were used instead (see Supplementary Material Appendix 2 for details of the patient-level data analysis of the ADNI).

A standardized Excel (Microsoft Corporation, Redmond, Washington, USA) document sheet was used for data extraction (for one special case of data extraction see Supplementary Material Appendix 3). Where data were available in graphical form only, we used an online software (Plot Digitizer) [19, 20]. The following data were extracted: source of data used in the studies (place of clinical trial or name of database), baseline characteristics of the population (age, gender, APOE status, and education level), type of exposure (Aβ, p-tau, and neurodegeneration), measurement technique of the exposure, data on cognitive impairment separately for the different exposure groups).

### Data synthesis

Generally, where several studies used the same population sample or cohort, only data from the study with the largest sample size were used. Conversion to Alzheimer’s dementia and to unspecified dementia was assessed together, as the definition of Alzheimer’s dementia varied between the studies, and the diagnosis was based on neurocognitive tests. If conversion to both types of dementia was given, the value of the conversion to unspecified dementia was used. The population with subjective cognitive symptoms was scored jointly with the CU population, as these subpopulations could not be differentiated objectively.

Odds ratio and hazard ratio values were used or calculated based on the available information (for details on the methodology, see Supplementary Material Appendix 4). Considering that studies report their results on different age groups, a meta-regression analysis was performed to investigate how age affects the likelihood of developing dementia based on Aβ levels.

Studies applied different analysis methods to identify Aβ positivity. Where multiple amyloid categories were being considered, the preferred method was amyloid PET. When relying on CSF analysis, the Aβ42/40 ratio was given precedence over Aβ42 since the 42/40 ratio has a higher concordance with amyloid PET [21]. To estimate the confounding effect caused by different amyloid measurement techniques a subgroup analysis was performed. For the assessment of p-tau, studies measured p-tau181 levels from CSF samples, or employed tau PET. While there is also a limited number of tau PET measurements in the ADNI, in order to ensure consistency in the analyses, we used exclusively the CSF p-tau181 levels from the ADNI database.

For the OR analysis, studies with varying follow-up times were pooled. To estimate the resulting bias, a meta-regression analysis was performed to explore how follow-up time affected the results.

### Statistical analysis

Statistical analyses were performed in the R programming environment (version 4.1.2) using the “meta” software package version 5.2-0. To visualize synthesized data, we used forest plots showing ORs or HRs and corresponding confidence intervals for each individual study and pooled effect sizes in terms of ORs and HRs. For dichotomous outcomes, odds ratios and hazard ratios with 95% confidence intervals (CI) were used as effect measures. To calculate odds ratios, the total number of patients in each study and the number of patients with the event of interest in each group were extracted from each study. Raw data from the selected studies were pooled using a random-effects model with the Mantel-Haenszel method [22–24]. The random-effects model was used as we assumed that the true effect would vary between studies due to differences in demographics and clinical measures, such as age or baseline cognitive impairment.

Heterogeneity was assessed by calculating I^2^, tau^2^, and the prediction interval. I^2^ is defined as the percentage of variability in the effect size that is not caused by sampling error, whereas tau^2^ is the square root of the standard deviation of the true effect size. As I^2^ is heavily dependent on the precision of the studies and tau^2^ is sometimes hard to interpret (as it is insensitive to the number of the studies and their precision), the prediction interval has also been calculated. The great advantage of the prediction interval is that this measure is easy to interpret: if the interval does not include zero, further studies are expected to show a similar result.

#### Sensitivity analysis

We performed outlier detection according to Viechtbauer et al. [25]. A study is considered an outlier if the confidence interval of the study does not overlap with the confidence interval of the pooled effect. The idea behind is to detect effect sizes that differ significantly from the overall effect. As a sensitivity analysis, we repeated the analyses after removing any outliers and then we compared the pooled effects before and after the exclusion, in order to detect if outliers would have a substiantial impact on the overall effect.

### Risk of bias assement

The risk of bias was assessed according to the recommendation of the Cochrane Collaboration; using the QUIPS tool [26], two investigators (ZH and YS) independently assessed the quality of the studies, and a third author solved disagreements. Publication bias was examined using the Peter’s regression test [27] and visual inspection of the adjusted Funnel-plots.

## Results

### Search results

**Fig. 1:**
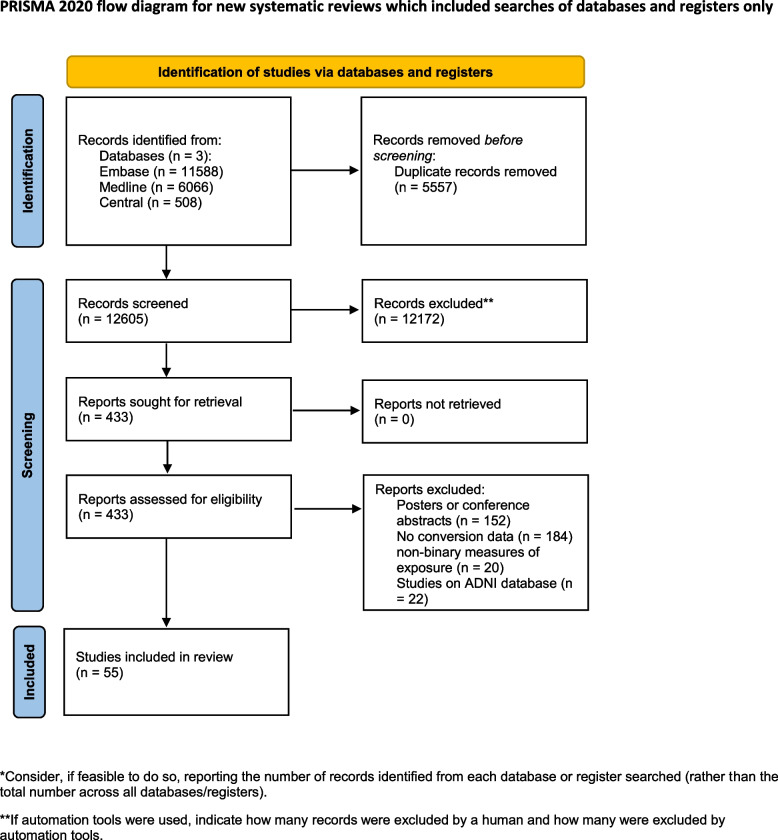
PRISMA flowchart of selection. Flowchart of the study screening process following the Preferred Reporting Items for Systematic Reviews and Meta-analysis (PRISMA) 2020 statement

During the systematic search (Fig. 1), 18,162 records were found, and finally, 46 eligible articles were obtained (Supplementary Material eTable 1); While some of the articles analyzed the same cohorts, we were able to pool data from 36 different cohorts or centres. The Cohens’s kappa was 0.91 for the title and abstract, and 0.86 for the full-text selection. Given the amount of data found, we decided to examine the targeted outcomes separately and focus only on the conversion data in this report.

The investigated studies expressed their results in different ways. They calculated unadjusted or adjusted hazard ratios or presented the number of conversions for the different follow-up periods. In the latter case, we calculated odds ratios for the defined time periods. The measured exposures also differed: data were given only for Aβ or in combination with p-tau or neurodegeneration. There were also differences in the techniques used to measure exposure, with CSF sample being used in some cases and PET scan in others.

During data extraction, one [28] article was excluded because of inconsistently reported conversion data, and four [15, 29–31] were excluded from the A/T analysis because the definition of the pathologic Aβ and p-tau was based on Aβ/p-tau ratio, which did not comply with the NIA-AA 2018 recommendation.

### Data synthesis

The eligible studies investigated three groups: CU, MCI, and mixed - in which the results were collectively expressed for both the MCI and CU groups. The CU group comprised either cognitively healthy subjects or individuals with only subjective cognitive complaints. To define the MCI group, all studies followed the Petersen criteria [32]. Four studies examined mixed groups. Since all of them studied large samples (*n*>180), it was considered more valuable to jointly analyze them with MCI, since the outcome was also the conversion to dementia. As a result of the joint analysis, our findings are based on a substantially larger sample. To support this decision, we performed a subgroup analysis comparing the Aβ positive MCI and mixed population studies. The OR differed significantly from the unexposed group in both the MCI (OR 5.83 [3.80; 8.93]) and the mixed (4.64 [95% CI 1.16; 18.61]) subgroups, and there was no significant difference between the two subgroups (*p*=0.55) (Supplementary Material eFigure 1).

#### Conversion from MCI to dementia

##### Aβ exposition - in OR

**Fig. 2:**
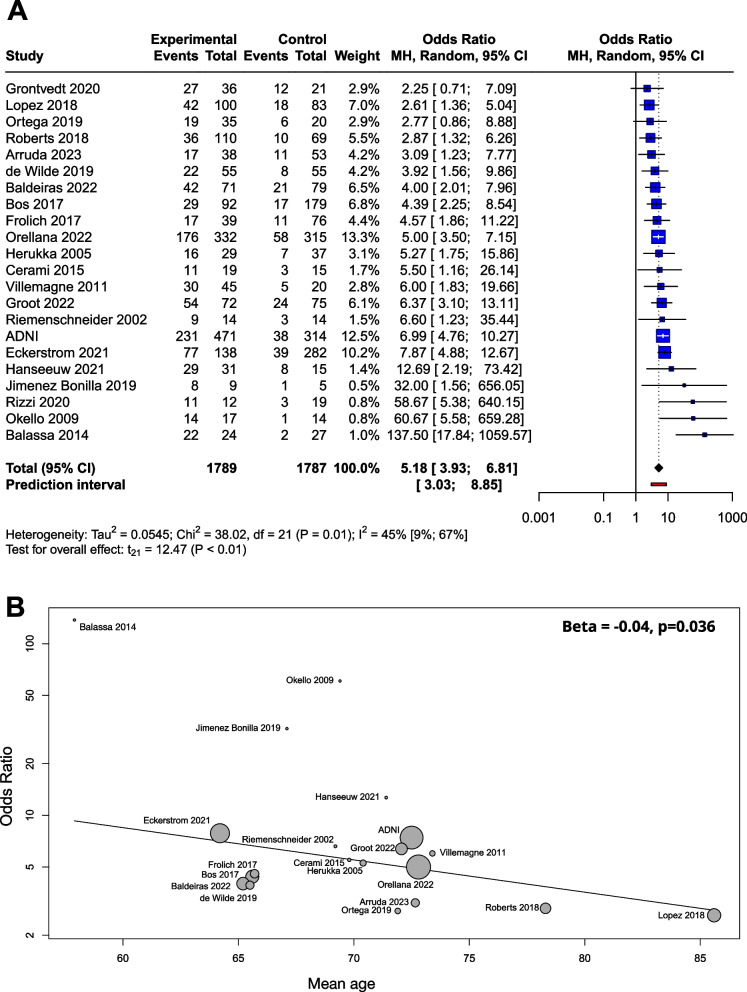
Conversion of Aβ exposed MCI groups to dementia in OR. The squares and bars represent the mean values and 95% CIs of the effect sizes, and the squares’ area reflects the weight of the studies. Diamonds represent the combined effects, and the vertical dotted line represents the line of no association. **A** OR for Aβ exposition; **B** meta-regression of age and ORs for conversion regarding Aβ exposure. The size of the circle is proportional to the weight of each study in the meta-analysis. The line corresponds to meta-regression with age as covariate, and beta represents the slope of ORs by mean age

Based on a mixed model meta-analysis of 3,576 subjects (Table 1), we observed a significant association between Aβ positivity and higher conversion rates. Compared to the unexposed, the OR for conversion to dementia in the amyloid positives were 5.18 [95% CI 3.93; 6.81]; t(21)=12.47; (*p*<0.0001). The I^2^- test for heterogeneity revealed that 44.8% of the variance across studies was due to heterogeneity (Fig. 2A). As a result of the outlier detection we excluded the Balassa study and found a very similar overall effect and a reduced heterogeneity (5.05 [95% CI 3.98; 6.40]; t(20) = 14.2; *p* < 0.0001; I^2^ = 31.4%). Meta-regression analysis of mean age showed a statistically significant decrease in OR values with increasing age (R^2^ = 59.05%, beta = -0.04, SE = 0.019, [95% CI = -0.03 to -0.083], df = 18, t = -2.27, *p* = 0.036) (Fig. 2B). The Hartunk-Knapp method was applied to adjust test statistics and confidence intervals to reduce the risk of false positives.

**Table 1:** Articles used for Aβ OR analyses in the Mild Cognitive Impairment (MCI) group

Study	Centre/cohort	Population	Subjects (*n*)	Age (mean (SD), or median (range))	Measurement technique (cut-offs)	Follow-up (month)		Conversion to
						Mean (SD)	Median (range)	
ADNI	ADNI	MCI	785	72.5 (7.5)	_amyloid_PET (SUVR >1.11), CSF Aβ42 (<977 pg/mL)	57 (40)	48	unspecified dementia
Arruda, 2023 [33]	Florida Alzheimer’s Disease Research Center	MCI	91	72.7 (8.7)	_amyloid_PET (v.r.^a^)	22.9 (7.1)	n.d.	unspecified dementia
Balassa, 2014 [15]	Hospital Clinic Barcelona, Spain	MCI	51	57.9 (6)	CSF Aβ42 (<500 pg/mL)	31 (15.8)	31.6 (8 - 82)	unspecified dementia
Baldeiras, 2022 [34]	Coimbra University Hospital; Hospital de Braga; Unidade Local de Saude de Matosinhos; Centro Hospi- ´ talar Baixo Vouga; Hospital Egas Moniz; Hospital de Faro, Portugal	MCI	150	65.2 (8.7)	CSF Aβ42/40 ratio (<0.068)	n.d.^b^	n.d. (12-50)	unspecified dementia
Bos, 2017 [35]	Alzheimer Center Limburg, LeARN, DESCRIPA cohort	Mixed^d^ (56.5% MCI)	271	65.6 (7.7)	CSF Aβ42 (≤ 550 pg/ml)	30 (14.4)	n.d.	unspecified dementia
Cerami, 2015 [36]	San Raffael Inst. Milan, Italy	MCI	34	69.8 (5.7)	CSF Aβ42 (<515 pg/m)	29 (8.5)	29 (15 - 60)	unspecified dementia
de Wilde, 2019 [37]^c^	Alzheimer Center and Department of Neurology, VU University Medical Center Amsterdam, Netherland	MCI	110	65.5 (7.5)	_amyloid_PET (v.r.), CSF Aβ42 (<813 pg/mL)	n.d.	22.8 (13.2 - 32.4)	unspecified dementia
Eckerstrom, 2021 [38]	Goteborg MCI study	Mixed^d^ (58.1% MCI)	420	64.2 (7.3)	CSF Aβ42 (≤482 ng/L)	31.6 (19)	n.d.	unspecified dementia
Frolich, 2017 [39]	DCN (Dementia Competence Network, German multicenter cohort study)	MCI	115	65.7 (9.3)	CSF Aβ42 (<600 pg/ml)	25.5 (9.8)	n.d.	unspecified dementia
Grontvedt, 2020 [14]	Department of Neurology, Univ. Hosp. Trondheim, Norway	MCI	57	64 (53 - 79)	CSF Aβ42 (<630 pg/ml)	n.d.	108 (72 - 120)	unspecified dementia
Groot, 2022 [40]	Malmö University Hospital, Sweden	MCI	147	72.1 (7.7)	CSF Aβ42/40 ratio (<0.07)	59.0 (25.1)	n.d.	unspecified dementia
Hanseeuw, 2021 [41]	Neurology Department, Saint-Luc University Hospital, Belgium	MCI	46	71.4 (7.5)	_amyloid_PET (v.r.)	38.4 (15.6)	n.d.	unspecified dementia
Herukka, 2005 [42]	Neurologic Department at Kuopio University Hospital, Finland	MCI	66	70.4 (7.4)	CSF Aβ42 (<452 pg/mL)	n.d.	36 (6-144)	unspecified dementia
Jimenez Bonilla, 2019 [43]	Neurology, University Hospital ‘Marqués de Valdecilla’, University of Cantabria, Santander, Spain	MCI	14	67.1 (5.1)	_amyloid_PET (v.r.)	60	60	unspecified dementia
Lopez, 2018 [44]	Ginkgo biloba memory study (GEM [Ginkgo Evaluation of Memory] Study, USA	Mixed^d^ (19.1% MCI)	183	85.6 (2.9)	_amyloid_PET (SUVR >1.57)	68.4 (20.4)	n.d.	unspecified dementia
Okello, 2009 [45]	Imperial College Healthcare NHS Trust [London], The National Hospital for Neurology and Neurosurgery [London], St. Margaret’s Hospital [Epping, and Victoria Hospital [Swindon], Turku Hosp., UK and Finland	MCI	31	69.4 (7.9)	_amyloid_PET (v.r.)	36	36	unspecified dementia
Orellana, 2022 [46]	ACE Alzheimer Center Barcelona, Spain	MCI	647	72.8 (7.8)	CSF Aβ42/40 ratio (<0.069)	21 (10.8)	n.d.	unspecified dementia
Ortega, 2019 [47]	Hospital Santa Maria de Lleida, Spain	MCI	55	71.9 (6.7)	CSF Aβ42 (<450 pg/ml)	n.d.	24 (no inf.)	unspecified dementia
Riemenschneider, 2002 [48]	Department of Psychiatry, Pearth, Australia	MCI	28	69.2 (7.9)	CSF Aβ42 (<500 pg/mL)	18	18	unspecified dementia
Rizzi, 2020 [49]	Division of Geriatric Neurology, Neurology Service, Hospital de Clínicas de Porto Alegre, Rua Ramiro Barcelos, Brazil	MCI	31	67.4 (60 - 78)	CSF Aβ42 (<618.5 pg/mL)	60	60	AD dementia^e^
Roberts, 2018 [50]	MCSA (Mayo Clinic Study of Aging)	MCI	179	78.3 (7.4)	_amyloid_PET (SUVR >1.42)	45.6 (24)	n.d.	AD dementia^e^
Villemagne, 2011 [51]	Austin Health Memory Disorders Clinic, USA	MCI	65	73.4 (8.5)	_amyloid_PET (SUVR >1.5)	20 (3)	n.d.	unspecified dementia

^a^visually read

^b^no data

^c^See details of data extraction in Supplement, Appendix 3

^d^A combined population of MCI and CU subjects

^e^Deffinition for AD in Rizzi 2020: McKhann et al. 2011 [8], in Roberts et al. 2018: DSM IV (American Psychiatric Association. Diagnostic and Statistical Manual of Mental Disorders. 4th ed. Washington, DC: American Psychiatric Association; 1994), McKhann et al. 2011 [8], McKhann et al. 1984 [7]

Beta-amyloid was determined by CSF Aβ42, CSF Aβ42/40 ratio or amyloid PET. When the three groups were compared in a subgroup analysis, the OR was 5.87 (2.83; 12.19) for CSF Aβ42, 5.00 (3.31; 7.55) for CSF Aβ42/40 ratio, and 5.32 (2.53; 11.18) for amyloid PET. The difference between the subgroups was not significant (*p*=0.88) (Supplementary Material eFigure 2).

The meta-regression analysis performed to examine the role of follow-up time showed no association with respect to the ORs (R^2^ = 0%, beta = -0.002, SE = 0.07, [95% CI = -0.02 - 0.01], df = 11, *p* = 0.77) (Supplementary Material eFigure 3A).

We used a funnel plot to examine publication bias (Supplementary Material eFigure 4A). Most of the studies with large sample sizes lie close to the midline, which confirms that the pooled effect size seems valid. However, the visual inspection of the plot raised the possibility of some publication bias in two ways: (1) Studies in the bottom right corner of the plot have significant results despite having large standard errors (2) The absence of studies in the bottom left corner (blank area in the figure) may indicate that studies with nonsignificant results were not published. In order to quantify funnel plot asymmetry, the Peter’s regression test was applied. The test results were not significant (*t* = 1.7, df = 20, *p* = 0.11) so no asymmetry was proven in the funnel plot.

##### The effect of Aβ exposition in terms of HR

**Fig. 3:**
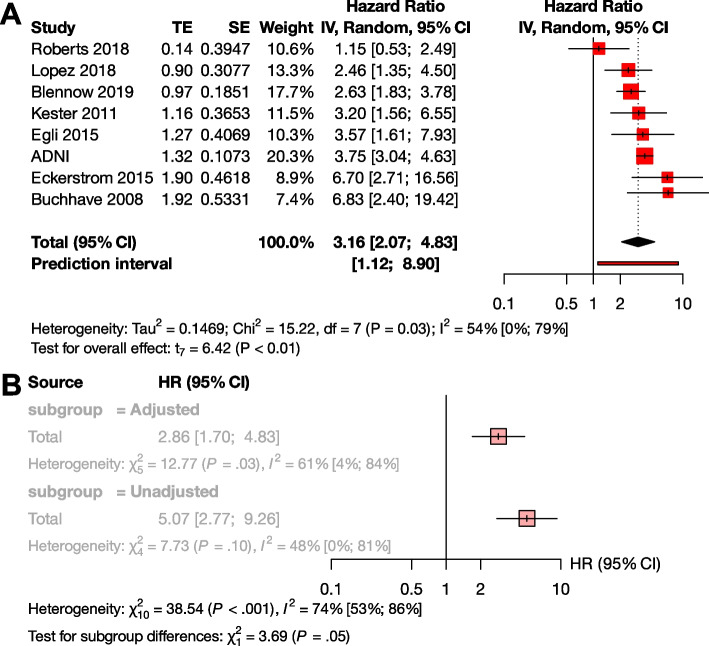
Conversion of Aβ exposed MCI groups to dementia in HR. The squares and bars represent the mean values and 95% CIs of the effect sizes, and the squares’ area reflects the weight of the studies. Diamonds represent the combined effects, and the vertical dotted line represents the line of no association.** A** HR for Aβ exposition; **B** sub-group analysis of studies with adjusted and unadjusted HR values

Several studies reported their results in HRs instead of or in addition to ORs (Supplementary Material eTable 2). The advantage of the HR value is that this measure is independent of the length of follow-up times of the studies. For these reasons, we also considered it important to analyze the results expressed in HR. Based on pooled data of patients studied (*n*=1,888), the HR for conversion to dementia was 3.16 [95% CI 2.07; 4.83], *p* < 0.001 (Fig. 3A).

To investigate the effect of adjustment, we conducted a subgroup analysis between the unadjusted and adjusted measurements. Although there was a trend for higher unadjusted HR values compared to the adjusted HRs, the difference did not reach statistical significance (unadjusted HR : 5.07 [95% CI 2.77 - 9.26], adjusted HR 2.86 [95% CI 1.70 - 4.83] *p*=0.055) (Fig. 3B). We could not analyze HR in the A+T-, A+T+, and A-T+ subgroups, due to the low number of available studies.

##### The effect of Aβ and p-tau exposition in terms of OR

**Fig. 4:**
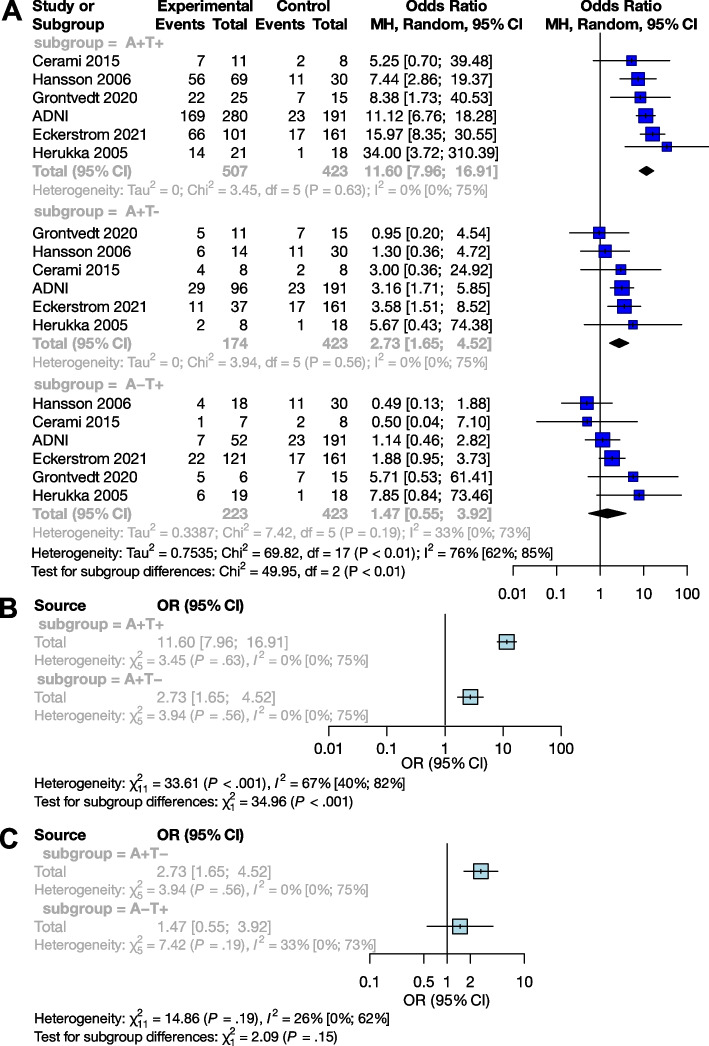
Conversion of Aβ and p-tau exposed MCI groups to dementia in OR. The squares and bars represent the mean values and 95% CIs of the effect sizes, and the squares’ area reflects the weight of the studies. Diamonds represent the combined effects, and the vertical dotted line represents the line of no association. **A** Aβ and p-tau expositions in OR; **B **sub-group analysis of comparisons between the A+T+ and A+T- groups; **C** sub-group analysis of comparisons between the A+T- and A-T+ groups

We examined the combined effect of p-tau and Aβ (Table 2), and compared A+T+, A+T-, and A-T+ exposures to A-T-. Based on pooled data for patients studied (n=1,327), the OR for conversion to dementia in A+T- was 2.73 [95% CI 1.65; 4.52], and the odds ratio was significantly higher in the presence of both exposures (A+T+) (*p*<0.001), with an OR of 11.60 [95% CI 7.96; 16.91]. The effect of A-T+ exposure on conversion was not significant (OR: 1.47 [0.55; 3.92]) (Fig. 4A).

**Table 2:** Articles used for Aβ and p-tau OR analyses in the Mild Cognitive Impairment (MCI) group

MCI population A+T+ vs. A-T-							
Study	Centre/cohort	Population	Measurement techniques (cut-offs)	Subjects (*n*.)	Age (mean (SD) / median (range))	Follow-up time (months)	
						Mean (SD)	Median (range)
ADNI	ADNI	MCI	_amyloid_PET (SUVR >1.11), CSF Aβ42 (<977 pg/mL); CSF p-tau181 (>23 pg/mL)	535	72.5 (7.5)	53 (38)	42
Cerami, 2015 [36]	San Raffael Inst. Milan, Italy	MCI	CSF Aβ42 (<515 pg/m); CSF p-tau181 (> 52.5 pg/mL)	19	69.8 (5.7)	29 (8.5)	29 (15-60)
Eckerström, 2021 [38]	Goteborg MCI study	Mixed^a^ (55.0 % MCI)	CSF Aβ42 (≤482 ng/L); CSF p-tau181 (≥52 ng/L)	262	64.2 (8.6)	34.74 (25)	n.d.^b^
Grontvedt, 2020 [14]	Department of Neurology, Univ. Hosp. Trondheim, Norway	MCI	CSF Aβ42 (<630 pg/ml); CSF p-tau181 (>66 pg/mL)	40	64 (53 - 79)	n.d.	108 (72-120)
Hansson, 2006 [52]	Malmö University Hospital, Sweden	MCI	CSF Aβ42 (<530 ng/L); CSF p-tau181 (≥60 ng/L)	99	71.8 (50 - 87)	n.d.	62.4 (48-81.6)
Herukka, 2005 [42]	Neurologic Department at Kuopio University Hospital, Finland	MCI	CSF Aβ42 (<452 pg/mL); CSF p-tau181 (>70 pg/mL)	39	70.4 (8.2)	n.d.	36 (6-144)
MCI population A+T- vs. A-T-							
ADNI	ADNI	MCI	_amyloid_PET (SUVR >1.11), CSF Aβ42 (<977 pg/mL); CSF p-tau181 (>23 pg/mL)	323	72.5 (7.5)	53 (38)	42
Cerami, 2015 [36]	San Raffael Inst. Milan, Italy	MCI	CSF Aβ42 (<515 pg/m); CSF p-tau181 (> 52.5 pg/mL)	16	69.8 (5.7)	29 (8.5)	29 (15-60)
Eckerström, 2021 [38]	Goteborg MCI study	Mixed ^a^ (44.4 % MCI)	CSF Aβ42 (≤482 ng/L); CSF p-tau181 (≥52 ng/L)	198	62.6 (8.3)	31.6 (19)	n.d.
Grontvedt, 2020 [14]	Department of Neurology, Univ. Hosp. Trondheim, Norway	MCI	CSF Aβ42 (<630 pg/ml); CSF p-tau181 (>66 pg/mL)	26	64 (53 - 79)	n.d.	108 (72-120)
Hansson, 2006 [52]	Malmö University Hospital, Sweden	MCI	CSF Aβ42 (<530 ng/L); CSF p-tau181 (≥60 ng/L)	44	71.8 (50 - 87)	n.d.	62.4 (48-81.6)
Herukka, 2005 [42]	Neurologic Department at Kuopio University Hospital, Finland	MCI	CSF Aβ42 (<452 pg/mL); CSF p-tau181 (>70 pg/mL)	26	70.4 (8.2)	n.d.	36 (6-144)
MCI population A-T+ vs A-T-							
ADNI	ADNI	MCI	_amyloid_PET (SUVR >1.11), CSF Aβ42 (<977 pg/mL); CSF p-tau181 (>23 pg/mL)	275	72.5 (7.5)	53 (38)	42
Cerami, 2015 [36]	San Raffael Inst. Milan, Italy	MCI	CSF Aβ42 (<515 pg/m); CSF p-tau181 (> 52.5 pg/mL)	15	69.8 (5.7)	29 (8.5)	29 (15-60)
Eckerström, 2021 [38]	Goteborg MCI study	Mixed ^a^ (46.1 % MCI)	CSF Aβ42 (≤482 ng/L); CSF p-tau181 (≥52 ng/L)	282	63.0 (7.6)	31.6 (19)	n.d.
Grontvedt, 2020 [14]	Department of Neurology, Univ. Hosp. Trondheim, Norway	MCI	CSF Aβ42 (<630 pg/ml); CSF p-tau181 (>66 pg/mL)	21	64 (53 - 79)	n.d.	108 (72-120)
Hansson, 2006 [52]	Malmö University Hospital, Sweden	MCI	CSF Aβ42 (<530 ng/L); CSF p-tau181 (≥60 ng/L)	48	71.8 (50 - 87)	n.d.	62.4 (48-81.6)
Herukka, 2005 [42]	Neurologic Department at Kuopio University Hospital, Finland	MCI	CSF Aβ42 (<452 pg/mL); CSF p-tau181 (>70 pg/mL)	37	70.4 (8.2)	n.d.	36 (6-144)

^a^A combined population of MCI and CU subjects

^b^no data

Subgroup analyses showed that the A+T+ group had a significantly higher odds of conversion compared to the A+T- group (*p* <0.001), while the A+T- and A-T+ groups did not differ significantly (*p*=0.15) (Fig. 4B and C).

#### Conversion from CU to MCI or dementia

##### The effect of Aβ exposition in terms of OR

**Fig. 5:**
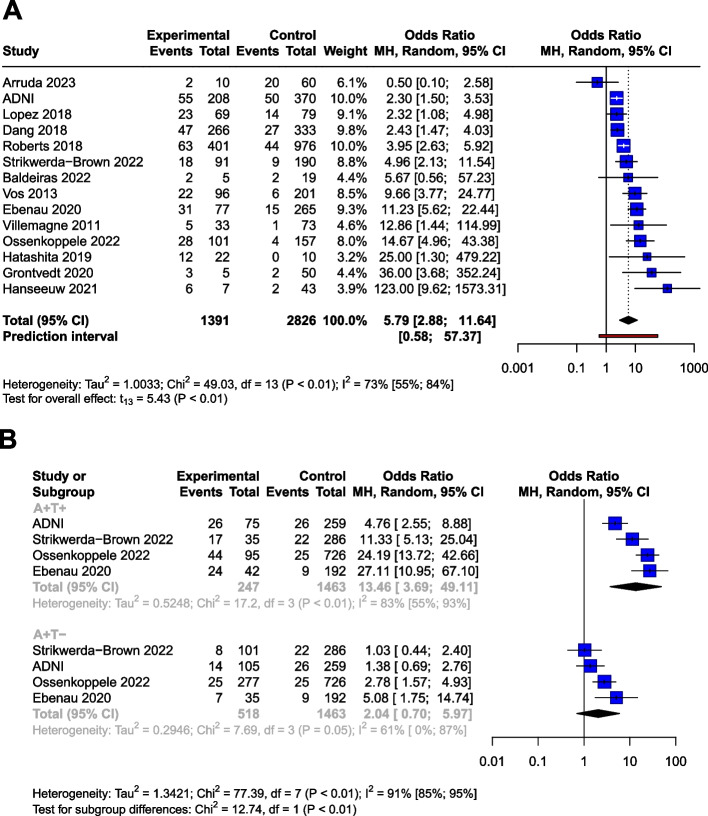
Conversion of Aβ and p-tau exposed CU groups to MCI or dementia in OR. The squares and bars represent the mean values and 95% CIs of the effect sizes, and the squares' area reflects the weight of the studies. Diamonds represent the combined effects, and the vertical dotted line represents the line of no association. **A** Aβ exposition in OR. **B** Aβ and p-tau expositions in OR

Analyses on the CU population (*n* = 4,217) yielded very similar results to the MCI sample. The OR for conversion to MCI or dementia was 5.79 [95% CI 2.88; 11.64] (t(13) = 5.43; *p* = 0.0001), the results of the studies did however show a high degree of heterogeneity (I^2^= 73% [55%; 84%]) (Table 3, Fig. 5A). As a result of the outlier detection we removed the Aruda study and found a very similar overall effect (6.33 [95% CI 3.42; 11.71]; t(12) = 6.54; *p* < 0.0001; I^2^ = 72.1%).

**Table 3:** Articles used for Aβ OR analyses in the Cognitively Unimpaired (CU) group

Study	Centre/cohort	Subjects (*n*)	Age (mean (SD), or median (range))	Measurement technique (cut-offs)	Follow-up (months)		Conversion to
					Mean (SD)	Median (range)	
ADNI	ADNI	578	72.9 (6.3)	_amyloid_PET (SUVR >1.11), CSF Aβ42 (<977 pg/mL)	69 (48)	54	MCI or unspecified dementia
Arruda, 2023 [33]	Florida Alzheimer’s Disease Research Center	70	70.2 (6.5)	_amyloid_PET (v.r.)	22.9 (7.1)	n.d.	MCI or unspecified dementia
Baldeiras, 2022 [34]	Coimbra University Hospital; Hospital de Braga; Unidade Local de Saude de Matosinhos; Centro Hospi- ´ talar Baixo Vouga; Hospital Egas Moniz; Hospital de Faro, Portugal	24	63.6 (8.9)	CSF Aβ42/40 ratio (<0.068)	n.d.	n.d. (12-50)	MCI or unspecified dementia
Dang, 2018 [53]	AIBL (The Australian Imaging, Biomarker & Lifestyle Flagship Study of Ageing)	599	70 (60 - 80)	_amyloid_PET (SUVR >1.40)	66.9	88.5	MCI or unspecified dementia
Ebenau, 2020 [54]	ADC (Amsterdam Dementia Cohort, SCIENCe! Subjective Cognitive Impairment Cohort)	342	60.0 (9.0)	_amyloid_PET (v.r.), CSF Aβ42 (<<813 pg/mL)	36 ( 24)	n.d.	MCI or unspecified dementia
Grontvedt, 2020 [14]	Department of Neurology, Univ. Hosp. Trondheim, Norway	55	68 (53 - 79)	CSF Aβ42	n.d.	108 (72 - 120)	unspecified dementia
Hanseeuw, 2021 [41]	Neurology Department, Saint-Luc University Hospital, Belgium	50	71.4 ( 7.5)	_amyloid_PET (v.r.)	38.4 (15.6)	n.d.	unspecified dementia
Hatashita, 2019 [55]	Department of Neurology, Shonan-Atsugi Hospital, Atsugi, Japan	32	71.0 (5.9)	PET (SUVR >1.39)	72 (21.6)	n.d.	MCI or unspecified dementia
Lopez, 2018 [44]	Ginkgo biloba memory study (GEM [Ginkgo Evaluation of Memory] Study, USA	148	84.2 (2.5)	_amyloid_PET (SUVR >1.57)	68.4 (20.4)	n.d.	MCI or unspecified dementia
Ossenkoppele, 2022 [56]	BioFINDER-1, -2	258	68.8 (10.1)	_amyloid_PET (SUVR >1.03 in BioFINDER-1, -2)	41.8 (18.9)	n.d.	unspecified dementia
Roberts, 2018 [50]	MCSA (Mayo Clinic Study of Aging)	1377	70. 4 ( 8.8)	_amyloid_PET (SUVR >1.42)	43.2 (24)	n.d.	MCI or AD dementia
Strikwerda-Brown, 2022 [57]	Prevent AD, HABS (Harvard Aging Brain Study)	281	72.1 (6.0)	_amyloid_PET (24 Centiloids for global Aβ)	n.d.	32.7 (15.7 – 58.0)	MCI or unspecified dementia
Villemagne, 2011 [51]	Austin Health Memory Disorders Clinic, USA	106	73.1 (7.5)	_amyloid_PET (SUVR >1.5)	20	20	MCI
Vos, 2013 [58]	Knight Alzheimer’s Disease Research Center (KADRC) of the Washington University School of Medicine (WUSM)in St. Louis, USA	297	72.9 (6.0)	CSF Aβ42 (<459 pg/mL)	n.d.	38.4 (12 - 156)	MCI or unspecified dementia

Meta-regression analysis of mean age did not show a significant association with OR. (R^2^ = 8.22%, beta = -0.05, SE = 0.05, [95% CI = -0.17 – 0.7], df = 11, *t* =, *p* = 0.37).

Meta-regression analysis also showed no association between follow-up time and ORs (R^2^ = 0.35%, beta = -0.014, SE = 0.024, [95% CI = -0.07 - 0.04], df = 8, *p* = 0.58) (Supplementary Material eFigure 3B).

We applied a funnel plot to examine publication bias (Supplementary Material eFigure 4B).Most of the studies with large sample sizes lie close to the midline, which reaffirms the pooled effect size’s validity. In order to quantify funnel plot asymmetry, Peter’s regression test was applied. The test results were not significant (*t* = 0.9, df = 12, *p* = 0.31) indicating that no asymmetry was demonstrated in the funnel plot.

##### The effect of Aβ exposition in terms of HR

Four cohorts provided HRs for the CU population (*n*=2700) with one cohort (ADNI) representing the 55.3% of the total sample (weight: 78.5%) (Supplementary Material eTable 3). The pooled HR for conversion was 2.33 [95% CI 1.88; 2.88] (*p*=0.001) (Supplementary Material eFigure 5)

##### The combined effect of Aβ and p-tau exposition in terms of OR

Using data from a total of 2228 subjects, we investigated the effect of p-tau in combination with Aβ (Table 4) in the CU population. The OR for conversion is 2.04 [95% CI 0.70; 5.97] for A+T-, and 13.46 [95% CI 3.69; 49.11] for the A+T+, compared to the A-T- group The OR shows a trend level increased risk (t=2.1, *P*=0.12) for the A+T- group compared to the A-T- group.

**Table 4:** Articles used for Aβ and p-tau OR analyses in the Cognitively unimpaired (CU) group

CU population A+T+ vs. A-T-						
Study	Centre/cohort	Measurement technique (cut-offs)	Subjects (*n*.)	Age (mean (SD) / median (range))	Follow-up time (months)	
					Mean (SD)	Median (range)
ADNI	ADNI	_amyloid_PET (SUVR >1.11), CSF Aβ42 (<977 pg/mL); CSF p-tau181 (>23 pg/mL)	334	72.9 (6.3)	69 (48)	54
Ebenau, 2020	ADC (Amsterdam Dementia Cohort, SCIENCe! Subjective Cognitive Impairment Cohort)	_amyloid_PET (v.r.), CSF Aβ42 (<<813 pg/mL); CSF p-tau181 (>52 pg/mL)	216	60.0 (9.0)	36 (24)	n.d.^a^
Ossenkoppele, 2022	BioFINDER-1, BioFINDER-2, HABS	_amyloid_PET (SUVR >1.03 in BioFINDER-1, -2, DVR >1.2 (>26 CL) in HABS); _tau_PET (SUVR >1.26 in BioFINDER-1, SUVR >1.34 in BioFINDER-2, SUVR >1.36 in HABS)	821	70.5 (9.8)	41.8 (18.9)	n.d.
Strikwerda-Brown, 2022	AIBL, Knight ADRC, Prevent AD	_amyloid_PET (24 Centiloids for global Aβ); _tau_PET (SUVR >1.27 for tau meta-ROI)	326	70.9 (5.6)	n.d.	39.8 (15.2 – 68.0)
CU population A+T- vs. A-T-						
ADNI	ADNI	_amyloid_PET (SUVR >1.11), CSF Aβ42 (<977 pg/mL); CSF p-tau181 (>23 pg/mL)	364	72.9 (6.3)	69 (48)	54
Ebenau, 2020	ADC (Amsterdam Dementia Cohort, SCIENCe! Subjective Cognitive Impairment Cohort)	_amyloid_PET (v.r.), CSF Aβ42 (<<813 pg/mL); CSF p-tau181 (>52 pg/mL)	227	60.0 (9.0)	36 (24)	n.d.
Ossenkoppele, 2022	BioFINDER-1, BioFINDER-2, HABS	_amyloid_PET (SUVR >1.03 in BioFINDER-1, -2, DVR >1.2 (>26 CL) in HABS); _tau_PET (SUVR >1.26 in BioFINDER-1, SUVR >1.34 in BioFINDER-2, SUVR >1.36 in HABS)	1003	70.5 (9.8)	41.8 (18.9)	n.d.
Strikwerda-Brown, 2022	AIBL, Knight ADRC, Prevent AD	_amyloid_PET (24 Centiloids for global Aβ); _tau_PET (SUVR >1.27 for tau meta-ROI)	387	70.9 (5.6)	n.d.	39.8 (15.2 – 68.0)

^a^no data

Similarly to the MCI population, subgroup analyses showed that the A+T+ group had significantly higher OR for conversion compared to the A+T- group (*p* <0.01). The analysis could not be performed for A-T+ due to the low number of these cases.

### Risk of bias assessment

The risk of bias was assessed separately for the analyses discussed above. The overall risk of the studies ranged from low to moderate, except in three cases: twice we found a high risk of bias due to attrition of above 50% [59, 60], and once due to a focus on monozygotic twins [61] (Supplementary Material, eFigure 6). These articles (*n*=197) were excluded from all analyses.

## Discussion

### Summary and context

A pathological Aβ state are strongly correlated with the risk of clinical progression. The odds ratio for conversion is 5.18 in the MCI population and 5.79 in the CU population. Therefore, measuring Aβ levels alone can identify a population at high risk. The OR for conversion to dementia differs significantly between the A+T+ and A+T- groups in both the MCI and CU populations: while the OR is 2.73 [95% CI 1.65; 4.52] for MCI and 2.04 [95% CI 0.70; 5.97] for CU subjects in the A+T- group, it increases to 11.60 [95% CI 7.96; 16.91] for MCI and 14.67 [95% CI 3.69; 49.11] for CU in the A+T+ group. Note that in the case of A+T- at CU population, only a trend-level statistical correlation is visible.

The results of the meta-regression show a decrease in OR with mean age (Fig. 2B). Based on this result it seems that the impact of Amyloid positivity on conversion is decreasing with age. The fact that age is a risk factor for dementia and vascular and other neurodegenerative damage are more frequent in elderly age is a possible explanation to this finding. Our findings combined with the results of Rodrigue et al. [62] suggests that amyloid burden increases with age, while its impact on conversion rates slightly decreases with age.

The appearance of Aβ is assumed to be one of the earliest signs of AD [63, 64]. Our results fit into this picture by showing that only the A+T+ and A+T- groups showed an increased risk for conversion compared to A-T-, the A-T+ group did not. Thus, Aβ alone is suitable for detecting the population at risk, while p-tau alone is not as effective in the prediction conversion. Our result is in line with previous studies showing that the A-T+ group has a weaker association with cognitive decline compared to the A+T- or A+T+ groups [65, 66]. However, it is important to emphasize that previous results showing that T+ status is closely associated with neurodegeneration and the A-T+ group is related to frontotemporal dementia [67]. More research is needed to fully explain the significance of the A-T+ group.

The PET scan is known to be a more sensitive tool for detecting Amyloid positivity compared to CSF sampling [68]. However, from a prognostic point of view, our results did not show a significant difference (*p*=0.73) between PET measurements (OR: 6.02) and the more cost-effective but invasive CSF Aβ42 measurements (OR: 5.11). It is important to note here that the present meta-analysis is underpowered for detecting prognostic differences between these methods. Due to the heterogeneity among studies, the impact of confounding factors, and standardised studies are required to evaluate the comparative prognostic value of these biomarkers accurately.

Our results based on ORs are further strengthened by the HR analyses giving similar results for Aβ exposure in the MCI (HR: 3.16) and CU (HR: 2.33) populations. It should be noted that in the HR analysis of the CU group, ADNI accounts for 78.5% of the weight, which is a limitation of this meta-analysis. This disproportionate representation may affect the overall result. Regarding the statistical trend-level association with a higher unadjusted HR, it should be noted that in the presence of a random distribution of other risk factors (e.g. baseline MMSE score or educational level), the unadjusted value may overestimate the HR. As in the case of a non-random distribution, the adjusted value underestimates the HR. With this in mind, we recommend reporting both values in the future.

Our analyses were performed on CU and MCI populations. Including mixed populations with the MCI population was a practical simplification, as several studies with a large number of cases gave their results combining MCI subjects with CU subjects, and we aimed to answer the set of questions based on the largest population. To investigate the potential bias of this method, we performed subgroup analysis comparing the mixed and MCI populations, and the result was not significant. The Aβ OR based on the mixed-only group is 4.64 [95% CI 1.16; 18.61], and the OR calculated on the MCI-only studies is 5.83 [95% CI 3.80; 8.93]. Thus, the inclusion of the mixed population in the pool decreases the OR of the main analysis (5.21 [95% CI 3.93; 6.90]) slightly (Supplementary Material eFigure 1).

### Strengths and limitations

There are several limitations to consider when interpreting our results. The study populations differ in several aspects; for cognitive status, the population ranges from those with no cognitive symptoms through those with subjective cognitive symptoms (these two groups were considered CU) to MCI groups. Therefore, the distance from the cognitive state corresponding to MCI or dementia also varies. Due to the different cut-offs used in the studies, subjects with grey area scores may oscillate between A- and A+ groups, increasing heterogeneity. Our study could not examine the role of other risk factors such as education, cardiovascular status, obesity, diabetes, depression, social and physical activity [69], or genetic status [70, 71], which may also contribute to heterogeneity. Furthermore, there is a considerable heterogeneity by mean age, and our meta-regression analysis of MCI group showed a significant decreasing effect of mean age on ORs.

In the OR analysis of Aβ in the CU group, in the context of the outlier value of the Arruda study, the possibility of a statistical extreme value can be assumed due to the small number of A+ subjects and the much larger A- group. Similarly, in the case of the Grontvedt [14] and Hanseeuw [41] studies, which show exceptionally high values, the A+ and A- groups show a similar uneven distribution. Similarly, the outliers in the MCI amyloid OR analysis are also associated with small sample sizes. For the Aβ HR analysis in the CU group, the interpretability of the result is strongly influenced by one specific cohort (ADNI), which accounts for 78% of the overall weight. In the A+T+/A+T-/A-T+ analyses, no outliers were found in either the MCI or CU groups.

Furthermore, we note that although the Aβ OR analyses could be confirmed by also calculating the HRs, the inability to analyze the effect of p-tau on HR due to the low number of studies limits the completeness of the A/T analysis.

We pooled studies reporting AD-type dementia conversion and studies reporting conversion to unspecified dementia. This simplification was necessary because different studies defined Alzheimer’s dementia differently, generally considering the amnestic clinical symptoms rather than biomarkers.

The fact that the studies used different neuropsychology tests to define MCI may contribute to the heterogeneity in the pooled sample. Another contributing factor would be the heterogeneity in the definition of MCI, however among the studies in our pool, only one, by Riemschneider et al. [48] (sample size = 28), precedes the 2003 ‘Key Symposium’ [72] that transformed the MCI concept. All other studies were published subsequent to it. While MCI subgroups were deifned after the 2003 Symposium, the definition of MCI (objective cognitive impairment, essentially preserved general cognitive functioning, preserved independence in functional abilities) did not change afterwards. Furthermore, most of the studies pooled in the analyses were published after 2010.

Another source of heterogeneity is the relatively small sample size of some studies, leading to a higher variability of results. However, we thought that including studies with lower sample sizes was also important to get a complete picture.

It is essential to discuss the difference in the follow-up times between studies. The follow-up times ranged from 20 months to more than 10 years. Follow-up times were given in different ways, either as mean, median or up to a certain point. While naturally, the odds of conversion increase over time, our meta-regression analysis suggests that there is no significant difference in the odds ratios over (follow-up) time. The moderate heterogeneity of the studies also points in this direction. We also note here that hazard ratios independent of follow-up time showed similar results to OR analyses. Finally, yet importantly, we would like to point out that pathological protein changes can begin up to 20 years before the appearance of symptoms [6]. Such an extended follow-up is very difficult to carry out; therefore, all studies were shorter than that.

The results for Aβ are based on 7,793 individuals, and the combined analyses of Aβ and p-tau are based on data of over 3,500 individuals. Studies using CSF sampling or amyloid/tau PET to detect Aβ and p-tau were pooled together, despite using different kits and thresholds for positivity, contributing to the heterogeneity of results. This variation is acknowledged in Tables 1, 2, 3 and 4, where the cut-off values are provided. Previous large population studies have indicated that amyloid and tau PET scans exhibit slightly higher sensitivity compared to CSF sampling techniques [73, 74, 68]. Nonetheless, the concordance between these diagnostic methods remains substantial. Moreover, findings from prior research (Lee et al. [75], Toledo et al. [76], Palmqvist et al. [77]) demonstrating high concordance across different amyloid CSF and amyloid PET measurements suggest that the impact of methodological differences on heterogeneity may be limited, All techniques are recommended by the National Institute on Aging-Alzheimer’s Association (NIA-AA) [6] for measurement.

### Future directions

Conversion to Alzheimer’s disease could not be analyzed specifically, as most of the articles examining conversion either did not define Alzheimer’s disease or the definition was based on neuropsychological testing but not on biomarkers (i.e., Aβ and p-tau status were assessed only at baseline). According to the NIA-AA guideline [6] and our results, we recommend biomarker-based studies to assess conversion rates to Alzheimer’s disease.

## Conclusions

In view of the Aβ and p-tau status, the most endangered population can be identified before the appearance of cognitive symptoms or at least at a mild stage. While the significance of Aβ in conversion is clear, it appears that its ability to predict the onset decreases with age. If we consider the current therapeutic limitations and the importance of early prevention, we believe that the initiation of non-pharmacological and pharmacological treatments should be related to Aβ and p-tau status rather than cognitive status.

Identifying the most endangered population also makes research more effective. The efficacy of different dementia prevention approaches can be more accurately assessed by knowing the Aβ and p-tau status of the patient. As the population targeted by the interventions can be more homogeneous, the effectiveness can be measured more precisely by identifying the population most at risk of conversion.

### Supplementary Information

**Supplementary Material 1.**

## Data Availability

The datasets used and/or analysed during the current study are available from the corresponding author on reasonable request.

## Abbreviations

A-: Non-pathologic levels of beta-amyloid
A+: Pathologic levels of beta-amyloid
Aβ: Beta-amyloid
AD: Alzheimer’s disease
ADNI: Alzheimer’s Disease Neuroimaging Initiative
CI: Confidance interval
CU: Cognitively unimpaired
CSF: Cerebrospinal fluid
HR: Hazard ratio
MCI: Mild cognitive impairment
N-: Absence of neurodegeneration
N+: Presence of neurodegeneration
NIA-AA: National Institute on Aging Alzheimer’s Association
OR: Odds ratio
PET: Positron emission tomography
p-tau: Phosphorylated tau
T-: Non-pathologic levels of phosphorylated tau
T+: Pathologic levels of phosphorylated tau
