# Supplementary material for: Risk of conversion to mild cognitive impairment or dementia among subjects with amyloid and tau pathology: a systematic review and meta-analysis
Source: Alzheimers Res Ther. 2024 Apr 12;16:81. doi: 10.1186/s13195-024-01455-2 (PMC11015617; doi:10.1186/s13195-024-01455-2)
Supplement: Supplementary file 1 — Supplementary Material 1. [file 13195_2024_1455_MOESM1_ESM.docx]

**Supplementary Material**

Zsolt Huszár, Gábor Csukly et al.; Risk of conversion to mild cognitive impairment or dementia among subjects with amyloid and tau pathology: A systematic review and meta-analysis

**Table of Contents**

**Appendix 1**: Search keys

**Appendix 2**: ADNI - Alzheimer's Disease Neuroimaging Initiative

**Appendix 3**: Data extraction in case of de Wilde, 2019

**Appendix 4**: OR and HR pooling

**eFigure 1**: Subgroup analysis of studies with MCI subjects versus studies with mixed (MCI+CU) population

**eFigure 2**: Subgroup analysis of studies using PET versus CSF Aβ42/40 versus CSF Aβ42 to assess amyloid in MCI studies

**eFigure 3A**: Meta-regression analysis of follow-up time and ORs for MCI

**eFigure 3B**: Meta-regression analysis of follow-up time and ORs for CU

**eFigure 4A** Funnel plot for MCI Aβ meta-analysis

**eFigure 4B** Funnel plot for CU Aβ meta-analysis

**eFigure 5** Conversion of Aβ exposed CU group to MCI or dementia in HR

**eFigure 6**: Risk of bias assessment

**eTable 1**: List of all eligible articles

**eTable 2**: Articles used for the Aβ HR analyses in the MCI group

**eTable 3**: Articles used for the Aβ HR analyses in the CU group

**eTable 4**: PRISMA 2020 Checklist

**References**

**Appendix 1** Search keys

**MEDLINE (via PubMed)**:

(Amyloid or Aβ) AND (cerebrospinal fluid OR CSF OR PET OR positron emission tomography) AND (cognitive AND (impairment OR dysfunction OR decline))

**Cochrane Library (CENTRAL)**:

(Amyloid or Aβ) AND (cerebrospinal fluid OR CSF OR PET OR positron emission tomography) AND (cognitive AND (impairment OR dysfunction OR decline))

**Embase**:

(Amyloid or Aβ) AND (cerebrospinal fluid OR CSF OR PET OR positron emission tomography) AND (cognitive AND (impairment OR dysfunction OR decline))

**Appendix 2** ADNI - Alzheimer's Disease Neuroimaging Initiative

Data used in the preparation of this article were obtained from the Alzheimer’s Disease Neuroimaging Initiative (ADNI) database (adni.loni.usc.edu). The ADNI was launched in 2003 as a public-private partnership, led by Principal Investigator Michael W. Weiner, MD. The primary goal of ADNI has been to test whether serial magnetic resonance imaging (MRI), positron emission tomography (PET), other biological markers, and clinical and neuropsychological assessment can be combined to measure the progression of mild cognitive impairment (MCI) and early Alzheimer’s disease (AD). For up-to-date information, see www.adni-info.org.

**Acknowledgement**:

Data collection and sharing for this project was funded by the Alzheimer's Disease Neuroimaging Initiative (ADNI) (National Institutes of Health Grant U01 AG024904) and DOD ADNI (Department of Defense award number W81XWH-12-2-0012). ADNI is funded by the National Institute on Aging, the National Institute of Biomedical Imaging and Bioengineering, and through generous contributions from the following: AbbVie, Alzheimer’s Association; Alzheimer’s Drug Discovery Foundation; Araclon Biotech; BioClinica, Inc.; Biogen; Bristol-Myers Squibb Company; CereSpir, Inc.; Cogstate; Eisai Inc.; Elan Pharmaceuticals, Inc.; Eli Lilly and Company; EuroImmun; F. Hoffmann-La Roche Ltd and its affiliated company Genentech, Inc.; Fujirebio; GE Healthcare; IXICO Ltd.; Janssen Alzheimer Immunotherapy Research & Development, LLC.; Johnson & Johnson Pharmaceutical Research & Development LLC.; Lumosity; Lundbeck; Merck & Co., Inc.; Meso Scale Diagnostics, LLC.; NeuroRx Research; Neurotrack Technologies; Novartis Pharmaceuticals Corporation; Pfizer Inc.; Piramal Imaging; Servier; Takeda Pharmaceutical Company; and Transition Therapeutics. The Canadian Institutes of Health Research is providing funds to support ADNI clinical sites in Canada. Private sector contributions are facilitated by the Foundation for the National Institutes of Health (www.fnih.org). The grantee organization is the Northern California Institute for Research and Education, and the study is coordinated by the Alzheimer’s Therapeutic Research Institute at the University of Southern California. ADNI data are disseminated by the Laboratory for Neuro Imaging at the University of Southern California.

**ADNI sponsors**:

Data collection and sharing for this project was funded by the Alzheimer's Disease Neuroimaging Initiative (ADNI) (National Institutes of Health Grant U01 AG024904) and DOD ADNI (Department of Defense award number W81XWH-12-2-0012). ADNI is funded by the National Institute on Aging, the National Institute of Biomedical Imaging and Bioengineering, and through generous contributions from the following: AbbVie, Alzheimer’s Association; Alzheimer’s Drug Discovery Foundation; Araclon Biotech; BioClinica, Inc.; Biogen; Bristol-Myers Squibb Company; CereSpir, Inc.; Cogstate; Eisai Inc.; Elan Pharmaceuticals, Inc.; Eli Lilly and Company; EuroImmun; F. Hoffmann-La Roche Ltd and its affiliated company Genentech, Inc.; Fujirebio; GE Healthcare; IXICO Ltd.; Janssen Alzheimer Immunotherapy Research & Development, LLC.; Johnson & Johnson Pharmaceutical Research & Development LLC.; Lumosity; Lundbeck; Merck & Co., Inc.; Meso Scale Diagnostics, LLC.; NeuroRx Research; Neurotrack Technologies; Novartis Pharmaceuticals Corporation; Pfizer Inc.; Piramal Imaging; Servier; Takeda Pharmaceutical Company; and Transition Therapeutics. The Canadian Institutes of Health Research is providing funds to support ADNI clinical sites in Canada. Private sector contributions are facilitated by the Foundation for the National Institutes of Health (www.fnih.org). The grantee organization is the Northern California Institute for Research and Education, and the study is coordinated by the Alzheimer’s Therapeutic Research Institute at the University of Southern California. ADNI data are disseminated by the Laboratory for Neuro Imaging at the University of Southern California.

**Methods used in patient level data analyses in the ADNI database**

We used patient level data from the ADNI database to calculate HRs and ORs. We used the 18F-Florbetapir (AV45) PET data as default Amyloid measurement, where it was available. Florbetapir standardized uptake value ratio (SUVR) was created by averaging the four cortical regions and dividing it by the cerebellum as reference. According to the ADNI recommendation we applied the SUVR cutoff of 1.11 and used the whole cerebellum region as reference^1^ . In a previous study Clark et. al. (2012)^2^ showed that Florbetapir positivity using the same cutoff strongly correlated with post-mortem autopsy results. If PET data was not available, we used Amyloid 1-42 CSF measurements to maximize the size of the analysis sample. According to Hansson et al. (2018)^3^ we applied a cutoff of 977 pg/ml for Amyloid 1-42 measurements (Roche Elecsys Abeta42 CSF measures were used), since this cutoff value showed the highest agreement with Amyloid PET results (overall percent agreement was 87% (95%CI = 84.2-89.5%)). Subjects were defined as p-tau positive by CSF p-tau levels (INNO-BIA AlzBio3 CSF measures were used) were up to 23 pg/ml, since Shaw et al (2009)^4^ showed on autopsy-based Alzheimer cases that this cutoff has the best classification power. We used the 18F-Flortaucipir-tau-PET measurements only in the CU cohort, since in the MCI cohort all other studies were based on CSF measurements. Subjects were defined as tau positive when at least one Braak-stage Region of Interest (ROI) surpassed a pre-established cut-off of 1.3 SUVR^5-7^.

Hazard Ratio (HR) calculation in the ADNI database

In the ADNI database HRs were calculated from a Cox Proportional Hazard Model (PROC PHREG in SAS 9.4). Conversion to MCI or conversion to dementia were the dependent (predicted) variables in separate models, while Amyloid positivity applying the above-mentioned procedures and cutoffs served as predictor variables (unadjusted analyses). In case of the adjusted analyses, we also included age, gender, education, baseline MMSE score and APOE status as covariates.

Data tabulation for Odds Ratio (OR) calculation in the ADNI database

The last available follow-up datapoint for all subjects. The PROC Freq procedure was used (SAS 9.4) for data tabulation of events and non-events in the analysis groups (e.g., Amyloid positive vs. Amyloid negative). The resulting data was included in the pooled analysis of ORs reported in the manuscript.

**Comparison with other ADNI studies**

Previous studies analyzing the ADNI sample reported very similar HRs for Amyloid positivity in MCI (Dickerson et al 2013^8^ (HR (CSF, Abeta, n=154, MCI) = 3.66 vs 3.75 (our calculation for MCI)), Spencer et al 2019^9^ (HR (CSF, Abeta, n=185, MCI) = 3.5 (2.0-6.1)), and overall conversion rates (Kim et al 2023)^10^ (MCI conversion rate was 32.3% (108/334) vs 35.2% (our calculation)).

**Appendix 3**

Data extraction of de Wilde et al. 2019 data extraction

In this study, amyloid status was assessed in subjects using both PET and CSF Aβ42 measurement techniques by the authors. They divided their participants into a "concordant" (A+ or A- status based on both techniques) and a "discordant" (different classification based on the two methods) group and gave conversion rates accordingly. As data by PET or CSF Aβ42 are not available separately, the results for discordant subjects were excluded from our metaanalysis. This study was not included in the subgroup analysis comparing PET and CSF Aβ42 measurement techniques.

**Appendix 4** Odds Ratio (OR) and Hazard Ratio (HR) pooling

OR pooling

An odds ratio analysis was performed for the studies that provided detailed data on the conversion of exposed groups and the follow-up time; even if a study did not give the OR value, we calculated it from the data if it was possible. The following rules were applied when summarizing the data from the individual studies:

- If there was data for both MCI and mixed population in the same study, the data for MCI was used for the analysis.

- We followed the A/T/N classification system^11^ regarding the three potentially examined alterations in the studies (A+ for pathological A- for normal Aβ level, and similarly for T+/T- abnormal or nonabnormal p-tau, and N+/N- depending on the presence of neurodegeneration). The analyses were performed for Aβ only and for Aβ + p-tau exposures. Neurodegeneration was not included in the analysis because fewer data would have been available in each subgroup and because of the aspecific nature of the lesion. For Aβ, only results where other lesions did not subdivide the group were considered (e.g., if data were available for only the A+T+ group (or A+N+, or A+T+N+), the result was excluded from the Aβ analysis. Still, if data were available for A+T+ and A+T- too, the two A+ groups were combined, considered as A+ group, and compared with group A- calculated by combining A-T+ and A-T- groups (and all of this is similar in the case of A+N+, or A+T+N+)). This rule was also valid in the case of Aβ+ p-tau analysis (e.g. studies concerning A+T+N+ were excluded, or if data were available, they were handled together with A+T+N-, compared with A-T- calculated from A-T-N- and A-T-N+ groups.

- As mentioned above, comparisons were always made with the unexposed group (i.e. A+ vs A- and A+T+/A+T-/A-T+ vs A-T-)

- Regarding follow-up time, we only used data for one time point per study, the mean or median value, or the most prolonged time point if no mean/median data were available. The OR values of studies for different follow-up times were pooled together.

- A subgroup analysis was conducted between studies with a mean and median follow-up time longer and shorter than five years to investigate this confounding effect of different follow-up times.

- in case of the subgroup analysis comparing the effect of Amyloid and Tau we included only those articles where both Amyloid and Tau were both measured

HR pooling

The HR values were compared according to the following rules:

- Studies where A+ status was not given independently of T/N status were excluded from the analysis.

- The adjusted HR value was used if both adjusted and unadjusted values were given to avoid the confounding effect of other risk factors (such as age, sex, ApoE status, education level, and baseline MMSE score), even if adjustments differed between studies. Where there was data for more than one type of adjoint, the broader one was chosen.

- A subgroup analysis (**main article Figure 3 B**) was conducted to compare the adjusted and unadjusted values to assess the confounding effect, where both data types were available.

**eFigure 1** Subgroup analysis of studies with MCI subjects versus studies with mixed (MCI+CU) population

The squares and bars represent the mean values and 95% CIs of the effect sizes, and the squares' area reflects the weight of the studies. Diamonds represent the combined effects, and the vertical dotted line represents the line of no association.

**eFigure 2** Subgroup analysis of studies using PET, CSF Aβ42/40 ratio, or CSF Aβ42 to assess amyloid in MCI studies

The squares and bars represent the mean values and 95% CIs of the effect sizes, and the squares' area reflects the weight of the studies. Diamonds represent the combined effects, and the vertical dotted line represents the line of no association.

**eFigure 3** Meta-regression analysis to examine the role of follow-up time for MCI (A) and CU (B) groups

**A**

**B**

meta-regression of follow-up time and ORs for conversion regarding Aβ exposure. The size of the circle is proportional to the weight of each study in the meta-analysis. The line corresponds to meta-regression with follow-up time as covariate, and beta represents the slope of ORs by the follow-up time **A**: MCI group: (R^2^ = 0%, beta = -0.002, SE = 0.07, [95% CI = -0.02 - 0.01], df = 11, p = 0.77); **B:** CU group: (R^2^ = 8.22%, beta = -0.05, SE = 0.05, [95% CI = -0.17 – 0.7], df = 11, t =, p = 0.37).

**eFigure 4** Funnel plot for MCI (A) and CU (B) Aβ meta-analysis

**A**

The funnel plot shows odds ratios (x-axis) against standard errors (y-axis) for each study. Dashed black lines form a triangle representing the pooled odds ratio (central axis) and the 95% confidence interval. Grey dots represent individual studies. Symmetry in the distribution of the points indicates low publication bias, as assessed by Peter's regression test and visual inspection. In order to quantify funnel plot asymmetry, the Peter’s regression test was applied. The test results were not significant (t = 1.7, df = 20, p = 0.11) indicating that there is no asymmetry in the funnel plot.

**B**

The funnel plot shows odds ratios (x-axis) against standard errors (y-axis) for each study. Dashed black lines form a triangle representing the pooled odds ratio (central axis) and the 95% confidence interval. Grey dots represent individual studies. Symmetry in the distribution of the points indicates low publication bias, as assessed by Peter's regression test and visual inspection. In order to quantify funnel plot asymmetry, the Peter’s regression test was applied. The test results were not significant (t = 0.9, df = 12, p = 0.31) indicating that there is no asymmetry in the funnel plot.

**eFigure 5** Conversion of Aβ exposed CU group to MCI or dementia in HR

The squares and bars represent the mean values and 95% CIs of the effect sizes, and the squares' area reflects the weight of the studies. Diamonds represent the combined effects, and the vertical dotted line represents the line of no association.

**eFigure 6,.** Risk of bias assessment

- MCI Aβ OR


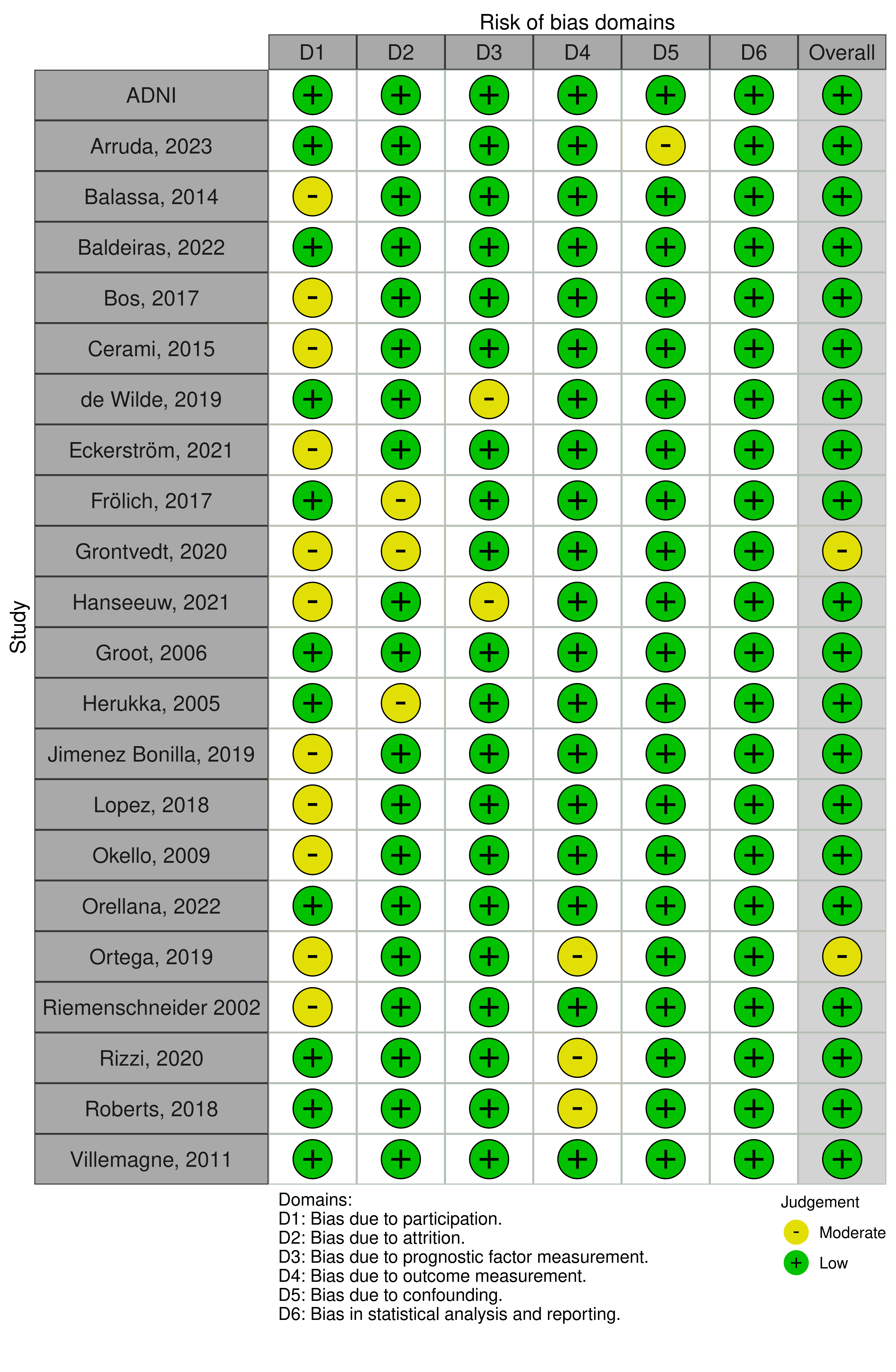

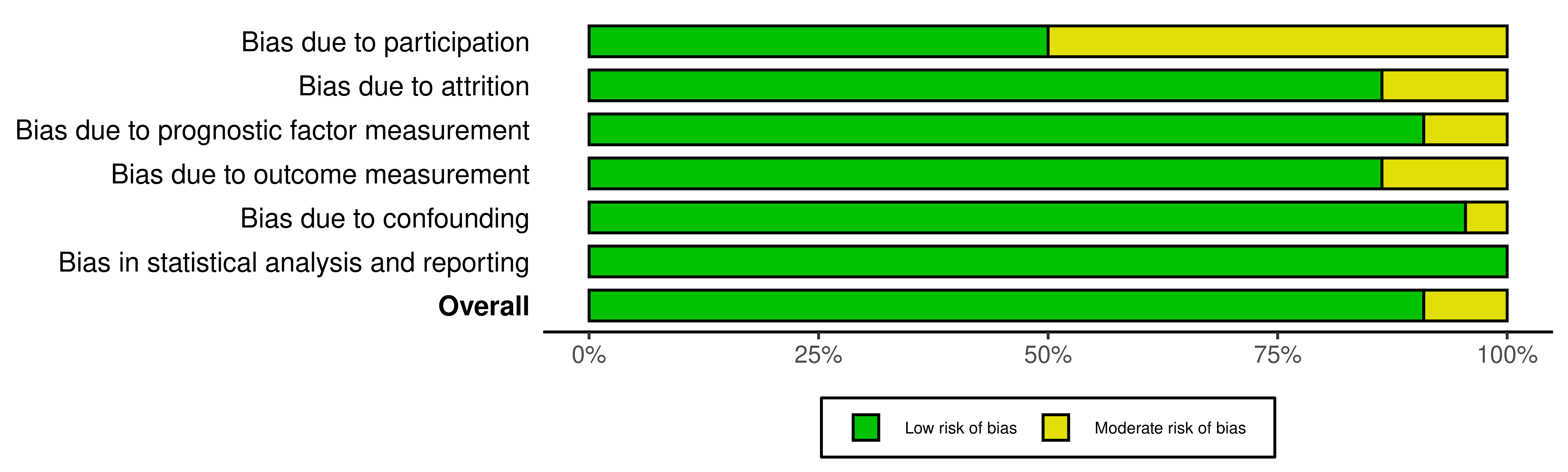


- MCI Aβ HR


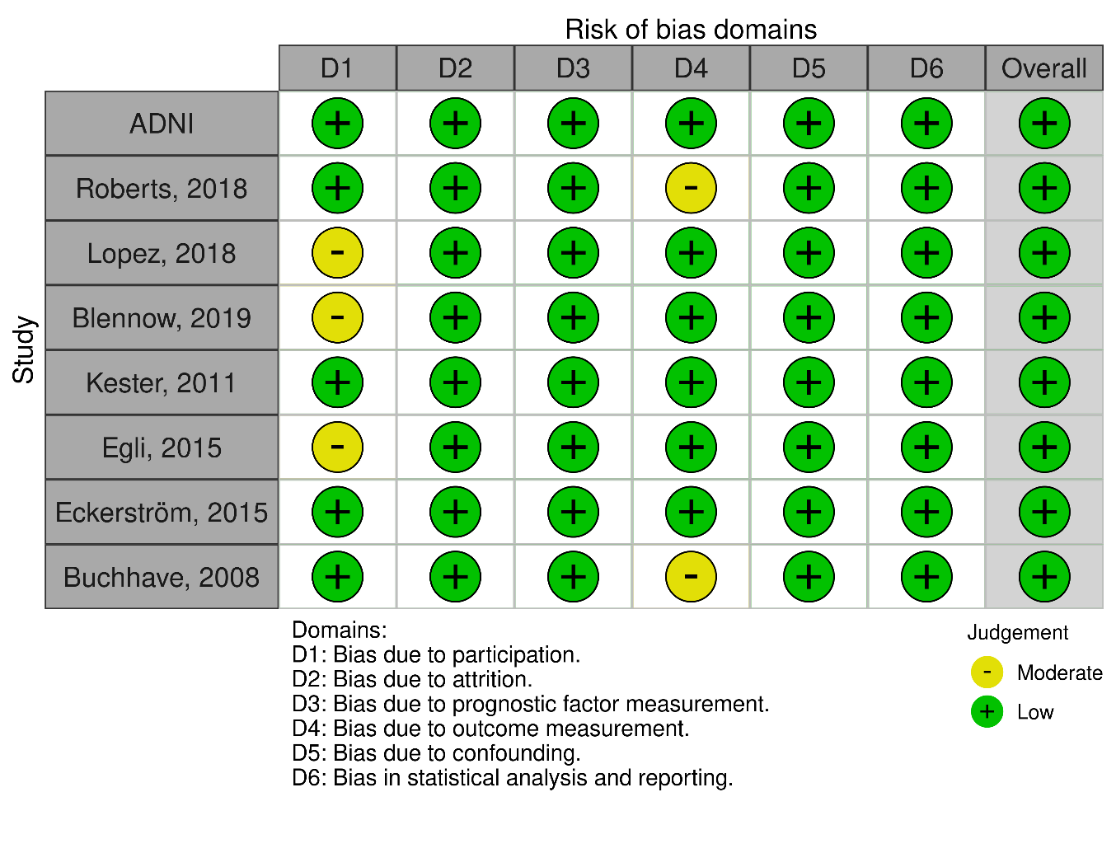

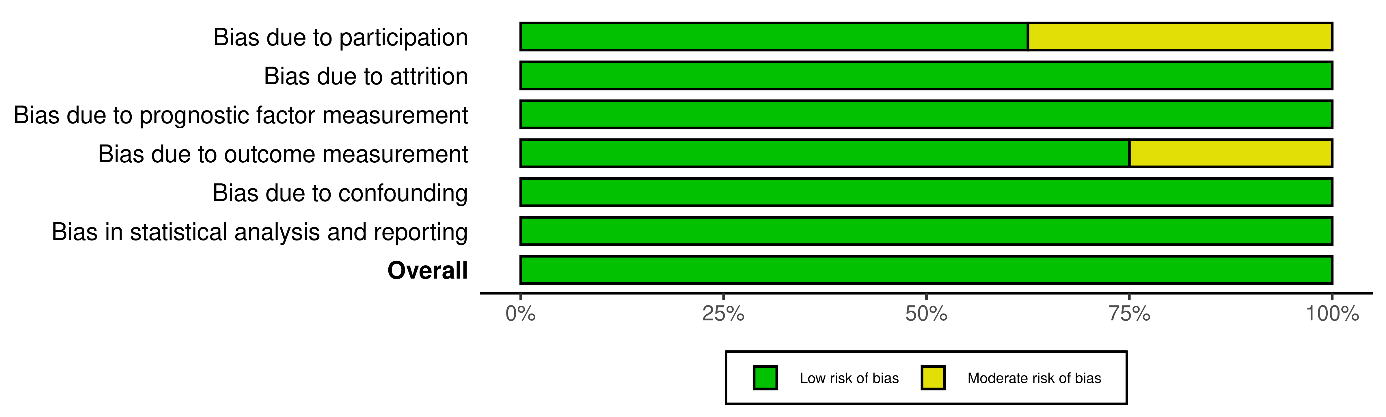


- MCI Aβ and p-tau OR


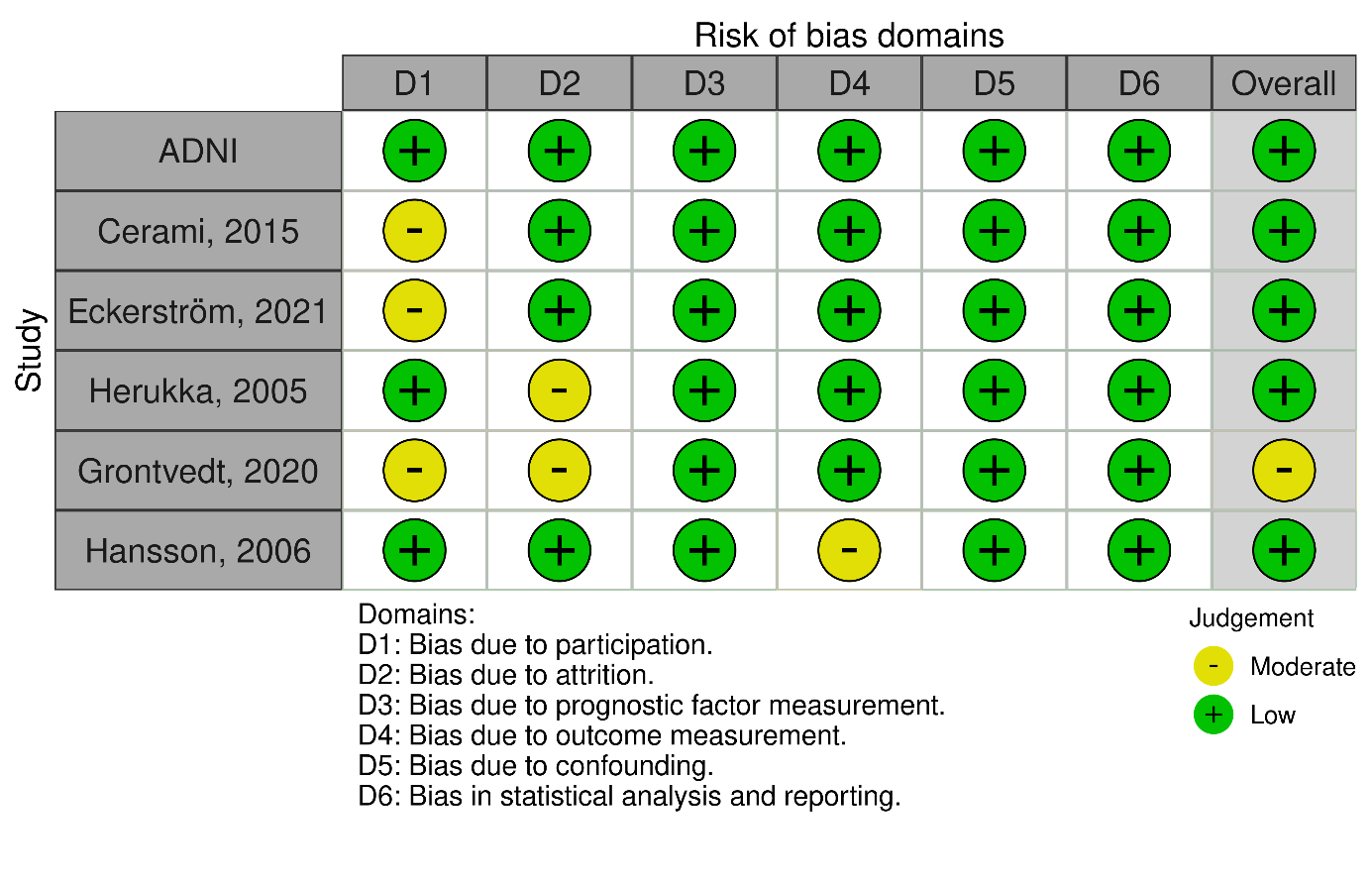


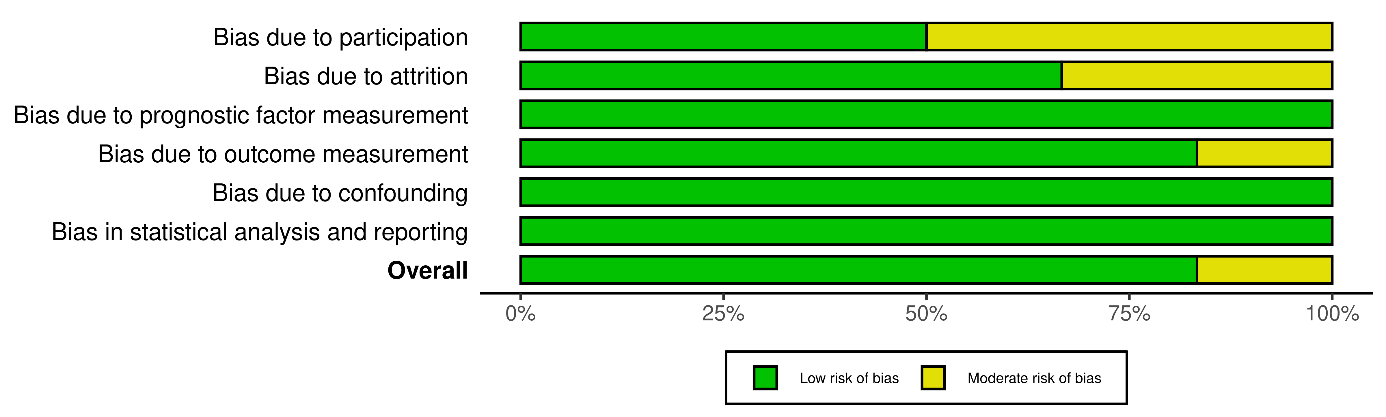


- CU Aβ OR


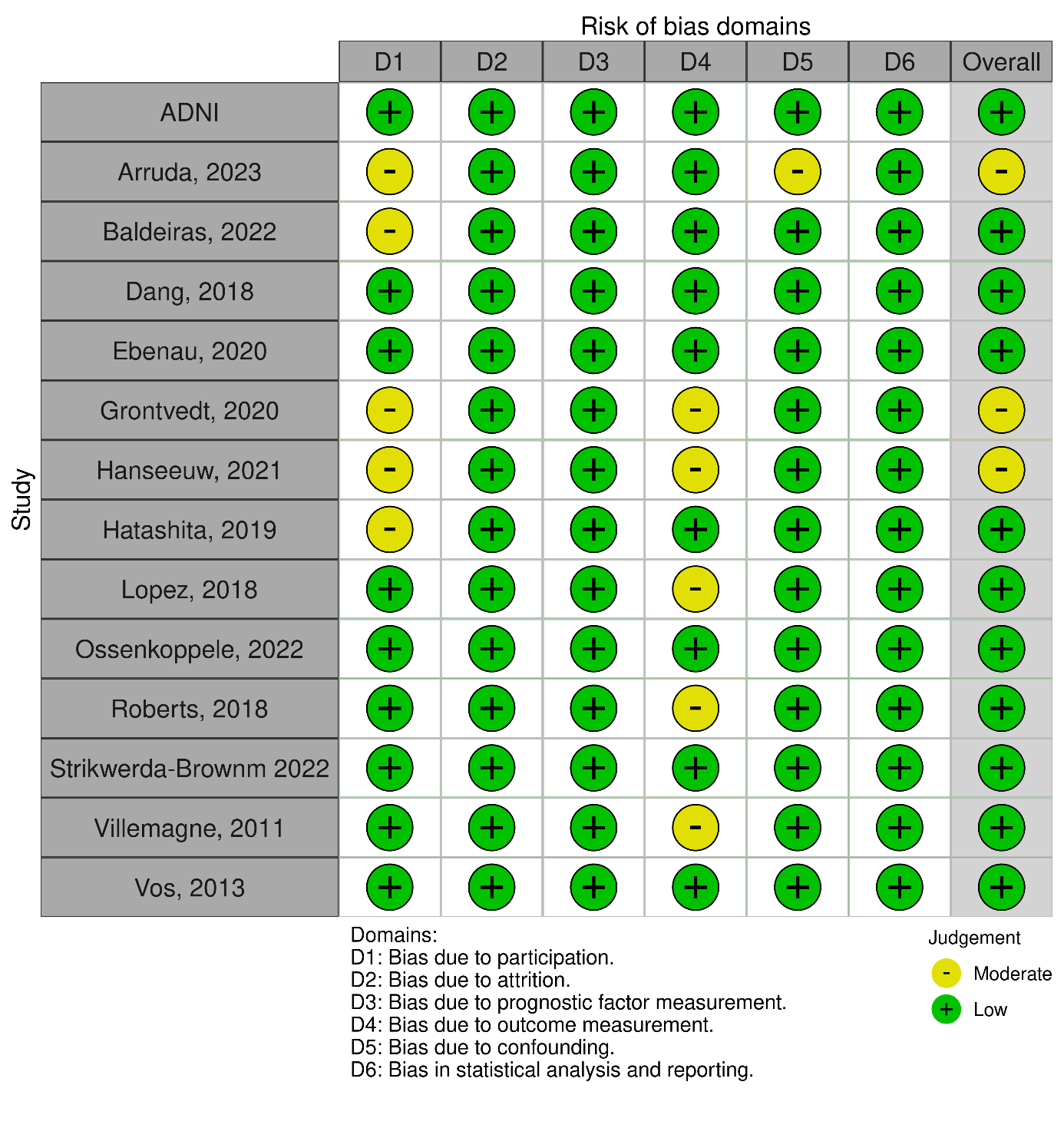


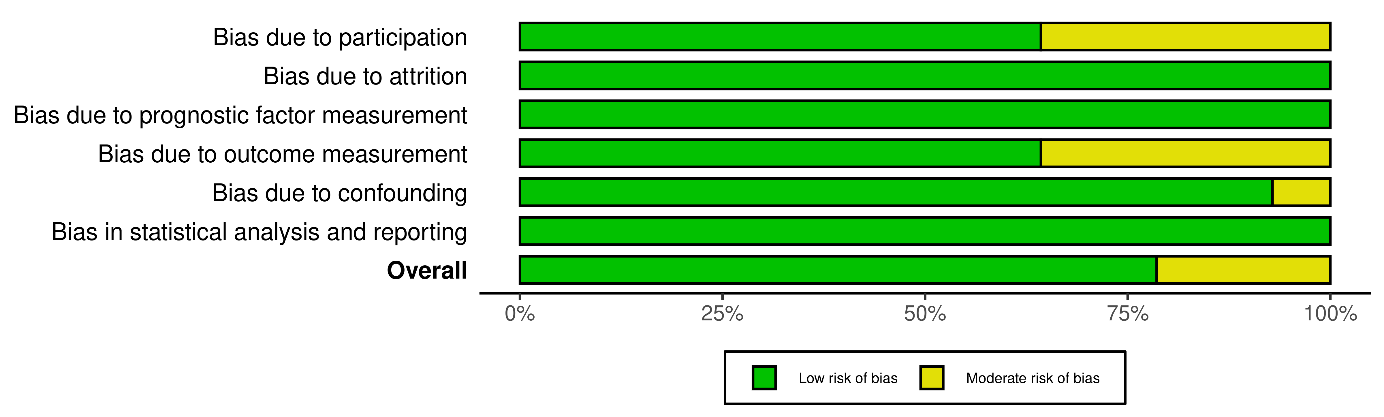


- CU Aβ and p-tau OR


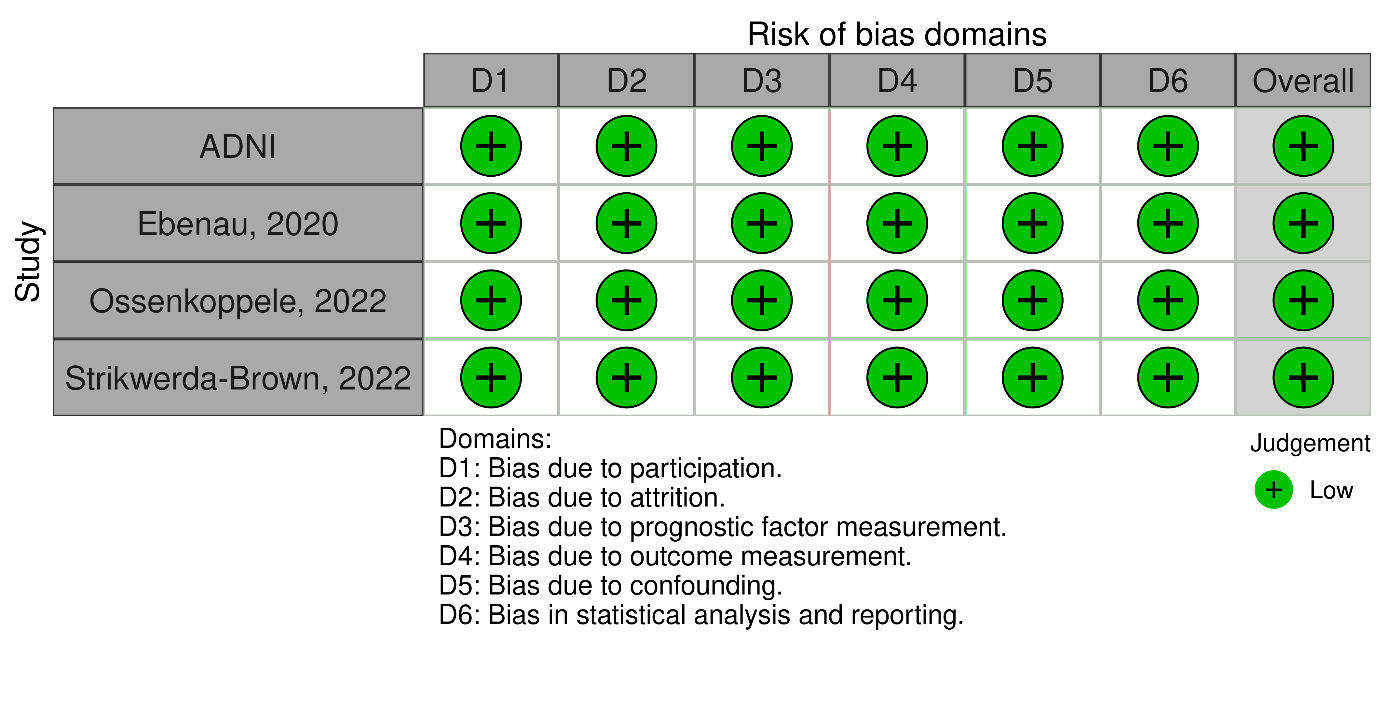


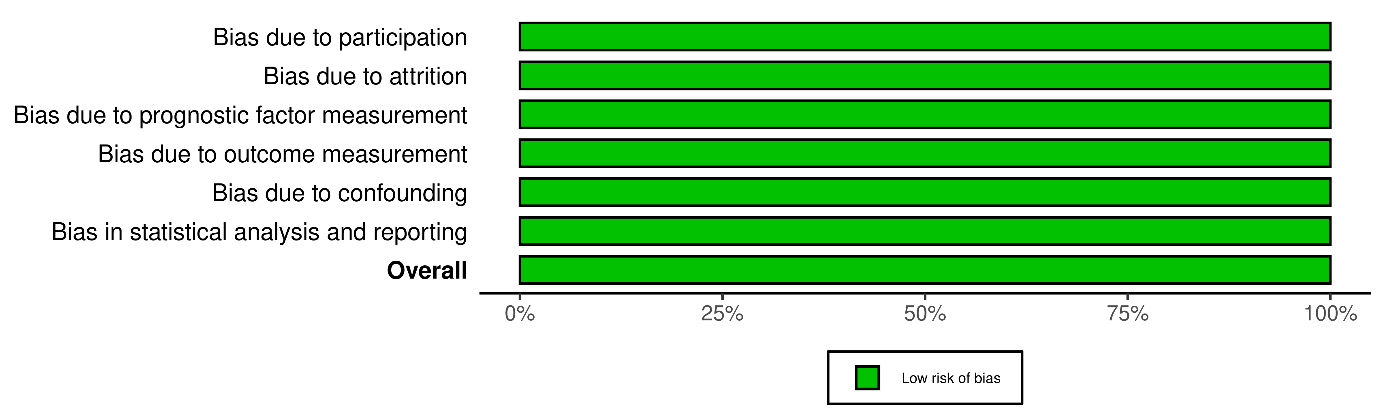


- CU Aβ HR


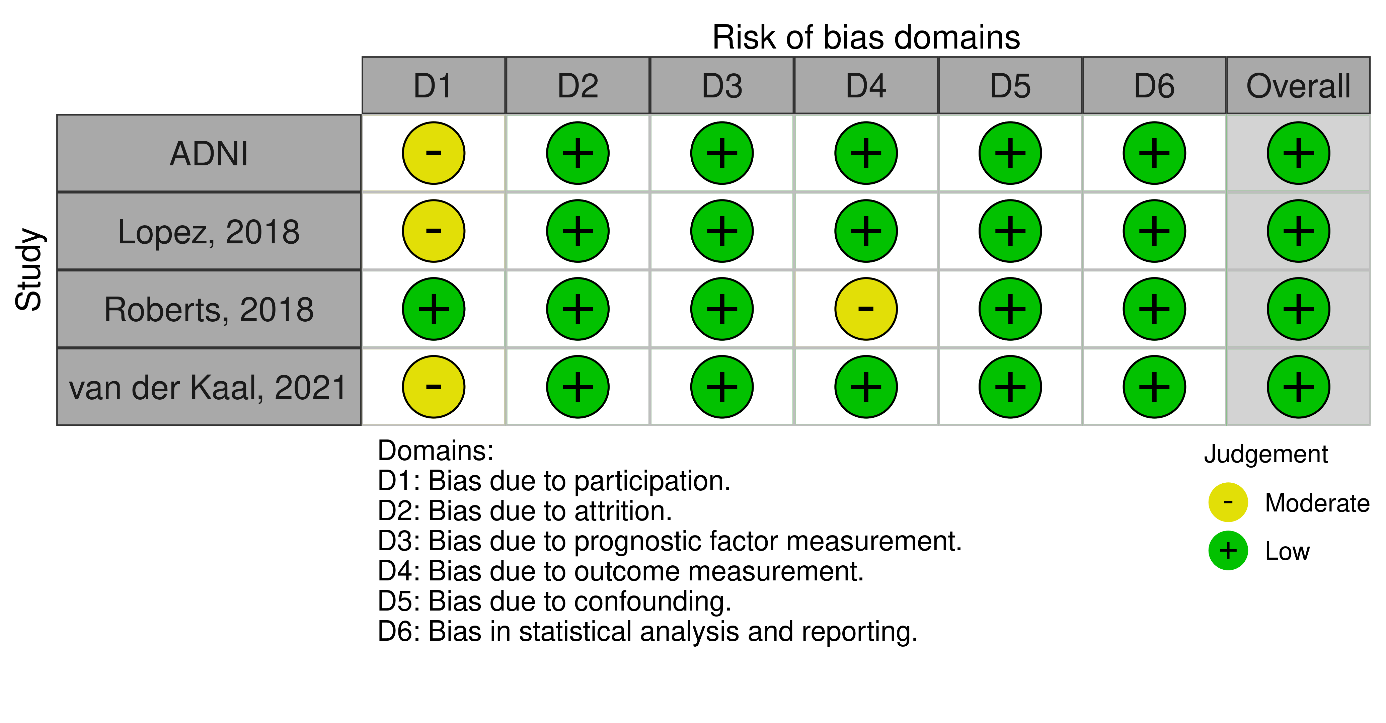

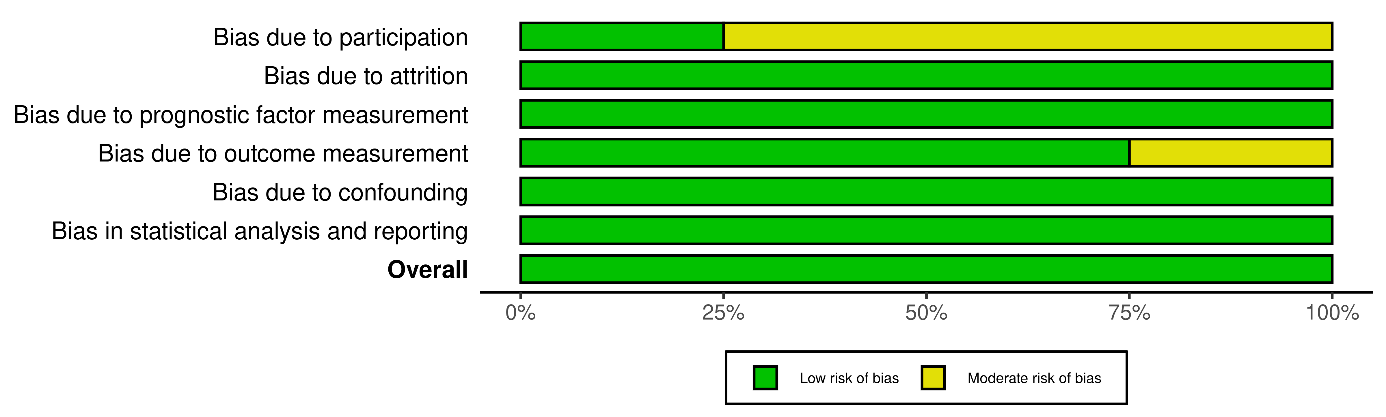


**eTable 1** List of all eligible articles

| study | centre/cohort | population | subjects (n) | investigated exposure(s) |
| --- | --- | --- | --- | --- |
| Arruda, 2022 | 1Florida Alzheimer’s Disease Research Center | CU, MCI | CU: 70  MCI: 91 | Aβ |
| Balassa, 2014 | Hospital Clinic Barcelona, Spain | MCI | 51 | Aβ, p-tau |
| Baldeiras, 2018 | Neurology Department of Coimbra University Hospital, Portugal | MCI | 56 | Aβ |
| Baldeires, 2022 | Coimbra University Hospital; Hospital de Braga; Unidade Local de Saude de Matosinhos; Centro Hospitalar Baixo Vouga; Hospital Egas Moniz; Hospital de Far, Portugal | CU, MCI | CU: 24  MCI: 150 | Aβ |
| Blennow, 2019 | BioFINDER study | mixed | 416 | Aβ, p-tau |
| Blom, 2009 | Department of Geriatrics at the Karolinska University Hospital in Huddinge Sweden | MCI | 28 | Aβ |
| Bos, 2017 | Alzheimer Center Limburg, LeARN, DESCRIPA cohort | mixed | 271 | Aβ |
| Buchhave, 2008 | Malmö University Hospital, Sweden | MCI | 114 | Aβ |
| Burnham, 2016 | AIBL (The Australian Imaging, Biomarker & Lifestyle Flagship Study of Ageing) | CU | 397 | Aβ |
| Buchhave, 2012 | Malmö University Hospital, Sweden | MCI | 134 | Aβ, p-tau |
| Cerami, 2015 | San Raffael Inst. Milan, Italy | MCI | 34 | Aβ, p-tau |
| Cerami, 2018 | San Raffael Inst. Milan, Italy | MCI | 30 | Aβ, p-tau |
| Dang, 2018 | AIBL | CU | 599 | Aβ |
| de Wilde, 2019 | Alzheimer Center and Department of Neurology, VU University Medical Center Amsterdam, Netherland | MCI | 110 | Aβ |
| Ebenau, 2020 | ADC ( Amsterdam Dementia Cohort), SCIENCe! Subjective Cognitive Impairment Cohort | CU | 342 | Aβ, p-tau |
| Eckerström, 2010 | Goteborg MCI study | MCI | 42 | Aβ |
| Eckerström, 2013 | Goteborg MCI study | mixed | 42 | Aβ |
| Eckerström, 2015 | Goteborg MCI study | MCI | 73 | Aβ |
| Eckerström, 2021 | Goteborg MCI study | mixed | 420 | Aβ, p-tau |
| Egli, 2015 | Memory Clinic, University Center for Medicine of Aging Basel, Felix Platter-Hospital, Basel, Switzerland | MCI | 36 | Aβ |
| Forlenza, 2015 | Institute of Psychiatry, University of Sao Paulo, Brazil | MCI | 41 | Aβ, p-tau |
| Frölich, 2017 | DCN (Dementia Competence Network, German multicenter cohort study) | MCI | 115 | Aβ |
| Grimmer, 2013 | Centre for Cognitive Disorders, Department of Psychiatry and Psychotherapy, Klinikum Rechts der Isar, Technische Universität München, Germany | MCI | 28 | Aβ |
| Grimmer, 2016 | Centre for Cognitive Disorders, Department of Psychiatry and Psychotherapy, Klinikum Rechts der Isar, Technische Universität München, Germany | MCI | 28 | Aβ |
| Grontvedt, 2020 | Department of Neurology, Univ. Hosp. Trondheim, Norway | MCI | 55 | Aβ, p-tau |
| Groot, 2022 | Memory Clinic at Skåne University Hospital in Malmö, Sweden | MCI | 147 | Aβ |
| Hanseeuw, 2021 | Neurology Department, Saint-Luc University Hospital, Belgium | CU, MCI | CU:50, MCI:46 | Aβ |
| Hansson, 2006 | Malmö University Hospital, Sweden | MCI | 131 | Aβ, p-tau |
| Hansson, 2007 | Malmö University Hospital, Sweden | MCI | 131 | Aβ |
| Hansson, 2009 | Malmö University Hospital, Sweden | MCI | 139 | Aβ, p-tau |
| Hatashita, 2019 | Department of Neurology, Shonan-Atsugi Hospital, Atsugi, Japan | CU | 32 | Aβ |
| Herukka, 2005 | Neurologic Department at Kuopio University Hospital, Finland | MCI | 66 | Aβ, p-tau |
| Hong, 2022 | Neurology, Uijeongbu St. Mary’s Hospital, The Catholic University of Korea, Seoul, Republic of Korea | CU | 47 | Aβ |
| Jimenez Bonilla, 2019 | Neurology, University Hospital ‘Marqués de Valdecilla’, University of Cantabria, Santander, Spain | MCI | 14 | Aβ |
| Kempainer, 2014 | Turku PET centre, Finnland | CU, MCI | CU:8, MCI:10 | Aβ |
| Kester, 2011 | Alzheimer Center and Department of Neurology, VU University Medical Center Amsterdam, Netherland | MCI | 100 | Aβ |
| Lopez, 2014 | Ginkgo biloba memory study (GEM [Ginkgo Evaluation of Memory] Study, USA | mixed | 146 | Aβ |
| Lopez, 2018 | Ginkgo biloba memory study (GEM [Ginkgo Evaluation of Memory] Study, USA | CU, mixed | CU: 148 mixed:183 | Aβ |
| Oberstein, 2022 | Clinic for Psychiatry and Psychotherapy, Nurnberg, Germany | mixed | 254 | Aβ, p-tau |
| Okello, 2009 | Imperial College Healthcare NHS Trust [London], The National Hospital for Neurology and Neurosurgery [London], St. Margaret’s Hospital [Epping, and Victoria Hospital [Swindon], Turku Hosp., UK and Finland | MCI | 31 | Aβ |
| Ong, 2015 | Austin Health Memory Disorders Clinic, USA | MCI | 45 | Aβ |
| Orellana, 2022 | ACE Alzheimer Center Barcelona, Spain | MCI | 647 | Aβ |
| Ortega, 2019 | Hospital Santa Maria de Lleida, Spain | MCI | 55 | Aβ |
| Ossenkoppele, 2022 | MCSA (Mayo Clinic Study of Aging), BioFINDER-1,-2, BACS (Berkeley Aging Cohort study), HABS (Harvard Aging Brain Study), AIBL, ADC | CU | 1325 | Aβ, p-tau |
| Prestia, 2015 | three independent European memory clinics** | MCI | 73 | Aβ |
| Riemenschneider, 2002 | Department of Psychiatry, Pearth, Australia | MCI | 28 | Aβ |
| Rizzi, 2020 | Division of Geriatric Neurology, Neurology Service, Hospital de Clínicas de Porto Alegre, Rua Ramiro Barcelos, Brazil | MCI | 31 | Aβ |
| Roberts, 2018 | MCSA | CU, MCI | CU: 1492 MCI:179 | Aβ |
| Salvadó, 2023 | BioFINDER-1, -2 | CU, MCI | CU: 1003  MCI: 475 | Aβ |
| Strikwerda-Brown, 2022 | Prevent-AD, HABS, AIBL, Knight ADRC (Knight Alzheimer’s Disease Research Center) | CU | 580 | Aβ, p-tau |
| Tomassen, 2022 | EMIF-AD Preclinic AD | CU | 122 | Aβ |
| van der Kaal, 2021 | AIBL (The Australian Imaging, Biomarker & Lifestyle Flagship Study of Ageing) | CU | 482 | Aβ |
| Villemagne, 2011 | Austin Health Memory Disorders Clinic, USA | CU, MCI | CU:106, MCI:65 | Aβ |
| Vos, 2012 | DESCRIPA cohort | MCI | 153 | Aβ |
| Vos, 2013 | Knight KADRC of the Washington University School of Medicine in St. Louis, USA | CU | 297 | Aβ |
| Wolk, 2018 | 28 clinical centers in the US and Europe *** | MCI | 229 | Aβ |

*: See details of data extraction in Supplement, Appendix 4.

**: Translational Outpatient Memory Clinic (TOMC) of the Scientific Institute for Research and Care of Alzheimer’s Disease, IRCCS Centro San Giovanni di Dio Fatebenefratelli in Brescia, Italy; the Alzheimer Center of the VU University Medical Center (VUMC), Amsterdam, The Netherlands; and the Memory Clinic, Department of Geriatric Medicine, Karolinska University Hospital Huddinge (KUHH), Stockholm, Sweden

­**­*: see at the original article: doi:10.1001/jamaneurol.2018.0894

**eTable 2** Articles used for the Aβ HR analyses in the MCI group

| study | centre/cohort | population | subjects (n) | age | conversion to | measurement technique | HR method | adjusted/unadjusted |
| --- | --- | --- | --- | --- | --- | --- | --- | --- |
| ADNI | ADNI | MCI | 787 | 72.5 (7.5) | dementia | PET, CSF Aβ42 | cox proportional | adjusted for age, sex, education level, baseline MMSE and APOE4 status and death as competing risk |
| Blennow, 2019 | BIOFINDER | mixed (54.1 % MCI) | 416 | 70 (5.6) | dementia | Aβ42 | cox proportional | adjusted for age, sex, years of education, baseline MMSE score |
| Buchhave, 2008 | Malmö University Hospital, Sweden | MCI | 114 | 70.8 (8.1) | AD^*^ | Aβ42 | cox proportional | adjusted for age sex, and APOE |
| Eckerström 2015 | Goteborg MCI study | MCI | 73 | 65.6 (3.9) | dementia | Aβ42 | cox proportional | unadjusted |
| Egli, 2015 | Memory Clinic, University Center for Medicine of Aging Basel, Felix Platter-Hospital, Basel, Switzerland | MCI | 36 | 69.6 (8) | dementia | Aβ42 | cox proportional | unadjusted |
| Kester, 2011 | Alzheimer Center and Department of Neurology, VU University Medical Center Amsterdam, Netherland | MCI | 100 | 67.8 (8.2) | dementia | Aβ42 | cox proportional | adjusted for age and sex |
| Lopez, 2018 | Ginkgo biloba memory study (GEM [Ginkgo Evaluation of Memory] Study, USA | mixed (19.1 % MCI) | 183 | 85.6 (2.9) | dementia | PET | cox proportional | adjusted for age and death as competing risk |
| Roberts, 2018 | MCSA (Mayo Clinic Study of Aging) | MCI | 179 | 78.3 (7.4) | AD* | PET | cox proportional | adjusted for age, sex, education level and APOE4 status |

*: Deffinition for AD in Buchhave 2008: McKhann et al. 1984^12^, in Roberts 2018: DSM IV (American Psychiatric Association. Diagnostic and Statistical Manual of Mental Disorders. 4th ed. Washington, DC: American Psychiatric Association; 1994), McKhann et al. 2011^13^, McKhann et al. 1984^12^

**eTable 3** Articles used for the Aβ HR analyses in the CU group

*: Deffinition for AD in van der Kaal 2021: McKhann et al. 1984^12^

| study | centre/cohort | subjects (n) | age | measurement technique | conversion to | HR method | adjusted/unadjusted | Hazard Ratio |
| --- | --- | --- | --- | --- | --- | --- | --- | --- |
| ADNI | ADNI | 578 | 72.9 (6.3) | PET, CSF Aβ42 | MCI or dementia | cox propotional | age sex and education and APOE status and baseline MMSE | 2.35 [1.96; 2.82] |
| Lopez, 2018 | Ginkgo biloba memory study (GEM [Ginkgo Evaluation of Memory] Study, USA | 148 | 84.2 (2.5) | PET | dementia | cox propotional | age and death as competing risk | 2.25 [1.10; 4.60] |
| Roberts, 2018 | MCSA (Mayo Clinic Study of Aging) | 1492 | 70. 4 ( 8.8) | PET | aMCI | cox propotional | age sex and education and APOE status | 2.10 [1.39; 3.17] |
| van der Kaal, 2021 | AIBL (The Australian Imaging, Biomarker & Lifestyle Flagship Study of Ageing) | 482 | 72 (6) | PET | MCI or AD^*^ | cox propotional | unadjusted | 6. 49 [1.41; 29.88] |

**eTable 4** PRISMA 2020 Checklist

| **Section and Topic** | **Item #** | **Checklist item** | **Location where item is reported (page no.)** |
| --- | --- | --- | --- |
| **TITLE** | | |  |
| Title | 1 | Identify the report as a systematic review. | 1 |
| **ABSTRACT** | | |  |
| Abstract | 2 | See the PRISMA 2020 for Abstracts checklist. | 2 |
| **INTRODUCTION** | | |  |
| Rationale | 3 | Describe the rationale for the review in the context of existing knowledge. | 3-4 |
| Objectives | 4 | Provide an explicit statement of the objective(s) or question(s) the review addresses. | 4-5 |
| **METHODS** | | |  |
| Eligibility criteria | 5 | Specify the inclusion and exclusion criteria for the review and how studies were grouped for the syntheses. | 6-7 |
| Information sources | 6 | Specify all databases, registers, websites, organisations, reference lists and other sources searched or consulted to identify studies. Specify the date when each source was last searched or consulted. | 5 |
| Search strategy | 7 | Present the full search strategies for all databases, registers and websites, including any filters and limits used. | Suppl. Mat. Appendix 1. |
| Selection process | 8 | Specify the methods used to decide whether a study met the inclusion criteria of the review, including how many reviewers screened each record and each report retrieved, whether they worked independently, and if applicable, details of automation tools used in the process. | 5-6 |
| Data collection process | 9 | Specify the methods used to collect data from reports, including how many reviewers collected data from each report, whether they worked independently, any processes for obtaining or confirming data from study investigators, and if applicable, details of automation tools used in the process. | 6-7 |
| Data items | 10a | List and define all outcomes for which data were sought. Specify whether all results that were compatible with each outcome domain in each study were sought (e.g. for all measures, time points, analyses), and if not, the methods used to decide which results to collect. | 6-7 and Suppl. Mat eTable 1 |
|  | 10b | List and define all other variables for which data were sought (e.g. participant and intervention characteristics, funding sources). Describe any assumptions made about any missing or unclear information. | 6-7 and Suppl. Mat eTable 1 |
| Study risk of bias assessment | 11 | Specify the methods used to assess risk of bias in the included studies, including details of the tool(s) used, how many reviewers assessed each study and whether they worked independently, and if applicable, details of automation tools used in the process. | 8-9 and Suppl. Mat. eFigure 6 |
| Effect measures | 12 | Specify for each outcome the effect measure(s) (e.g. risk ratio, mean difference) used in the synthesis or presentation of results. | 7-8 |
| Synthesis methods | 13a | Describe the processes used to decide which studies were eligible for each synthesis (e.g. tabulating the study intervention characteristics and comparing against the planned groups for each synthesis (item #5)). | 6 |
|  | 13b | Describe any methods required to prepare the data for presentation or synthesis, such as handling of missing summary statistics, or data conversions. | 6 and Suppl. Mat Appendix 3-4 |
|  | 13c | Describe any methods used to tabulate or visually display results of individual studies and syntheses. | 6-7 |
|  | 13d | Describe any methods used to synthesize results and provide a rationale for the choice(s). If meta-analysis was performed, describe the model(s), method(s) to identify the presence and extent of statistical heterogeneity, and software package(s) used. | 7-8 |
|  | 13e | Describe any methods used to explore possible causes of heterogeneity among study results (e.g. subgroup analysis, meta-regression). | 8 |
|  | 13f | Describe any sensitivity analyses conducted to assess robustness of the synthesized results. | 8 |
| Reporting bias assessment | 14 | Describe any methods used to assess risk of bias due to missing results in a synthesis (arising from reporting biases). | 8 and Suppl. Mat. eFigure 4 |
| Certainty assessment | 15 | Describe any methods used to assess certainty (or confidence) in the body of evidence for an outcome. | 7-8 |
| **RESULTS** | | |  |
| Study selection | 16a | Describe the results of the search and selection process, from the number of records identified in the search to the number of studies included in the review, ideally using a flow diagram. | 9 and  Figure 1 |
|  | 16b | Cite studies that might appear to meet the inclusion criteria, but which were excluded, and explain why they were excluded. | 9 and 31 |
| Study characteristics | 17 | Cite each included study and present its characteristics. | Table 1-3, Suppl. Mat. eTable 1-4. |
| Risk of bias in studies | 18 | Present assessments of risk of bias for each included study. | 31 and Suppl. Mat. eFigure 6. |
| Results of individual studies | 19 | For all outcomes, present, for each study: (a) summary statistics for each group (where appropriate) and (b) an effect estimate and its precision (e.g. confidence/credible interval), ideally using structured tables or plots. | Figures 2-5, Suppl. Mat. eFigure 1-3 and 5 |
| Results of syntheses | 20a | For each synthesis, briefly summarize the characteristics and risk of bias among contributing studies. | 10-31 and Suppl. Mat. eFigure 6 |
|  | 20b | Present results of all statistical syntheses conducted. If meta-analysis was done, present for each the summary estimate and its precision (e.g. confidence/credible interval) and measures of statistical heterogeneity. If comparing groups, describe the direction of the effect. | 10-31 and Figures 2-5, Suppl. Mat. eFigure 1-3 and 5 |
|  | 20c | Present results of all investigations of possible causes of heterogeneity among study results. | 10-31, Figure 2-4, eFigure 1-3 and 5 |
|  | 20d | Present results of all sensitivity analyses conducted to assess the robustness of the synthesized results. | Figures 2-5 and Suppl Mat. eFigure 1-5 |
| Reporting biases | 21 | Present assessments of risk of bias due to missing results (arising from reporting biases) for each synthesis assessed. | 31 and Suppl. Mat. eFigure 6. |
| Certainty of evidence | 22 | Present assessments of certainty (or confidence) in the body of evidence for each outcome assessed. | Figures 2-5. and Suppl. Mat. eFigure 1-5 |
| **DISCUSSION** | | |  |
| Discussion | 23a | Provide a general interpretation of the results in the context of other evidence. | 32-35 |
|  | 23b | Discuss any limitations of the evidence included in the review. | 34-35 |
|  | 23c | Discuss any limitations of the review processes used. | 34-35 |
|  | 23d | Discuss implications of the results for practice, policy, and future research. | 35-36 |
| **OTHER INFORMATION** | | |  |
| Registration and protocol | 24a | Provide registration information for the review, including register name and registration number, or state that the review was not registered. | 3, 5 |
|  | 24b | Indicate where the review protocol can be accessed, or state that a protocol was not prepared. | 5, 19 |
|  | 24c | Describe and explain any amendments to information provided at registration or in the protocol. | 5-6 |
| Support | 25 | Describe sources of financial or non-financial support for the review, and the role of the funders or sponsors in the review. | 39 |
| Competing interests | 26 | Declare any competing interests of review authors. | 39 |
| Availability of data, code and other materials | 27 | Report which of the following are publicly available and where they can be found: template data collection forms; data extracted from included studies; data used for all analyses; analytic code; any other materials used in the review. | 7-8, 39 |

*From:*  Page MJ, McKenzie JE, Bossuyt PM, Boutron I, Hoffmann TC, Mulrow CD, et al. The PRISMA 2020 statement: an updated guideline for reporting systematic reviews. BMJ 2021;372:n71. doi: 10.1136/bmj.n71

For more information, visit: <http://www.prisma-statement.org/>

1. Landau S, William J. Florbetapir processing methods. 2015

2. Clark CM, Schneider JA, Bedell BJ, et al. Use of florbetapir-PET for imaging beta-amyloid pathology. *Jama*. Jan 19 2011;305(3):275-83. doi:10.1001/jama.2010.2008

3. Hansson O, Seibyl J, Stomrud E, et al. CSF biomarkers of Alzheimer's disease concord with amyloid-β PET and predict clinical progression: A study of fully automated immunoassays in BioFINDER and ADNI cohorts. *Alzheimers Dement*. Nov 2018;14(11):1470-1481. doi:10.1016/j.jalz.2018.01.010

4. Shaw LM, Vanderstichele H, Knapik-Czajka M, et al. Cerebrospinal fluid biomarker signature in Alzheimer's disease neuroimaging initiative subjects. *Ann Neurol*. Apr 2009;65(4):403-13. doi:10.1002/ana.21610

5. Biel D, Brendel M, Rubinski A, et al. Tau-PET and in vivo Braak-staging as prognostic markers of future cognitive decline in cognitively normal to demented individuals. *Alzheimer's Research & Therapy*. 2021/08/12 2021;13(1):137. doi:10.1186/s13195-021-00880-x

6. Franzmeier N, Neitzel J, Rubinski A, et al. Functional brain architecture is associated with the rate of tau accumulation in Alzheimer’s disease. *Nature Communications*. 2020/01/17 2020;11(1):347. doi:10.1038/s41467-019-14159-1

7. Maass A, Landau S, Baker SL, et al. Comparison of multiple tau-PET measures as biomarkers in aging and Alzheimer's disease. *Neuroimage*. Aug 15 2017;157:448-463. doi:10.1016/j.neuroimage.2017.05.058

8. Dickerson BC, Wolk DA. Biomarker-based prediction of progression in MCI: Comparison of AD signature and hippocampal volume with spinal fluid amyloid-β and tau. *Front Aging Neurosci*. 2013;5:55. doi:10.3389/fnagi.2013.00055

9. Spencer BE, Jennings RG, Brewer JB. Combined Biomarker Prognosis of Mild Cognitive Impairment: An 11-Year Follow-Up Study in the Alzheimer's Disease Neuroimaging Initiative. *J Alzheimers Dis*. 2019;68(4):1549-1559. doi:10.3233/jad-181243

10. Kim YJ, Kim SE, Hahn A, et al. Classification and prediction of cognitive trajectories of cognitively unimpaired individuals. *Front Aging Neurosci*. 2023;15:1122927. doi:10.3389/fnagi.2023.1122927

11. Jack CR, Jr., Bennett DA, Blennow K, et al. NIA-AA Research Framework: Toward a biological definition of Alzheimer's disease. *Alzheimers Dement*. Apr 2018;14(4):535-562. doi:10.1016/j.jalz.2018.02.018

12. McKhann G, Drachman D, Folstein M, Katzman R, Price D, Stadlan EM. Clinical diagnosis of Alzheimer's disease: report of the NINCDS-ADRDA Work Group under the auspices of Department of Health and Human Services Task Force on Alzheimer's Disease. *Neurology*. Jul 1984;34(7):939-44. doi:10.1212/wnl.34.7.939

13. McKhann GM, Knopman DS, Chertkow H, et al. The diagnosis of dementia due to Alzheimer's disease: recommendations from the National Institute on Aging-Alzheimer's Association workgroups on diagnostic guidelines for Alzheimer's disease. *Alzheimers Dement*. May 2011;7(3):263-9. doi:10.1016/j.jalz.2011.03.005
